# Photocatalytic C(sp^3^) radical generation *via* C–H, C–C, and C–X bond cleavage

**DOI:** 10.1039/d2sc00202g

**Published:** 2022-04-18

**Authors:** Chia-Yu Huang, Jianbin Li, Chao-Jun Li

**Affiliations:** Department of Chemistry, FRQNT Centre for Green Chemistry and Catalysis, McGill University 801 Sherbrooke Street W. Montreal Quebec H3A 0B8 Canada cj.li@mcgill.ca

## Abstract

C(sp^3^) radicals (R˙) are of broad research interest and synthetic utility. This review collects some of the most recent advancements in photocatalytic R˙ generation and highlights representative examples in this field. Based on the key bond cleavages that generate R˙, these contributions are divided into C–H, C–C, and C–X bond cleavages. A general mechanistic scenario and key R˙-forming steps are presented and discussed in each section.

## Abstract 2

C(sp^3^) radicals (R˙) are of broad research interest and synthetic utility.

## Graphical abstract

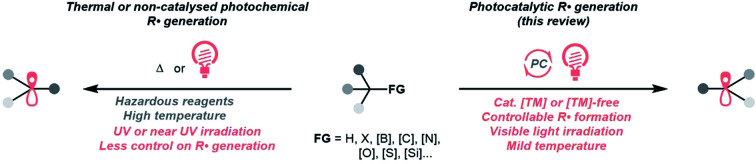

## 1. Introduction

The C(sp^3^) radical (R˙) represents one of the fundamental organic species in synthetic chemistry, which is highly enabling in various settings. R˙ can be derived from feedstock chemicals such as alkanes, alkenes, alcohols, amines, aldehydes, ketones, carboxylic acids and their derivatives, making it a versatile option for different synthetic purposes. Besides, it features complementary reactivities to other alkyl intermediates (*e.g.*, carbocation, carbanion, and carbene), providing flexible synthetic routes to build up the C(sp^3^)-rich scaffold and complexity.

**Scheme 1:**
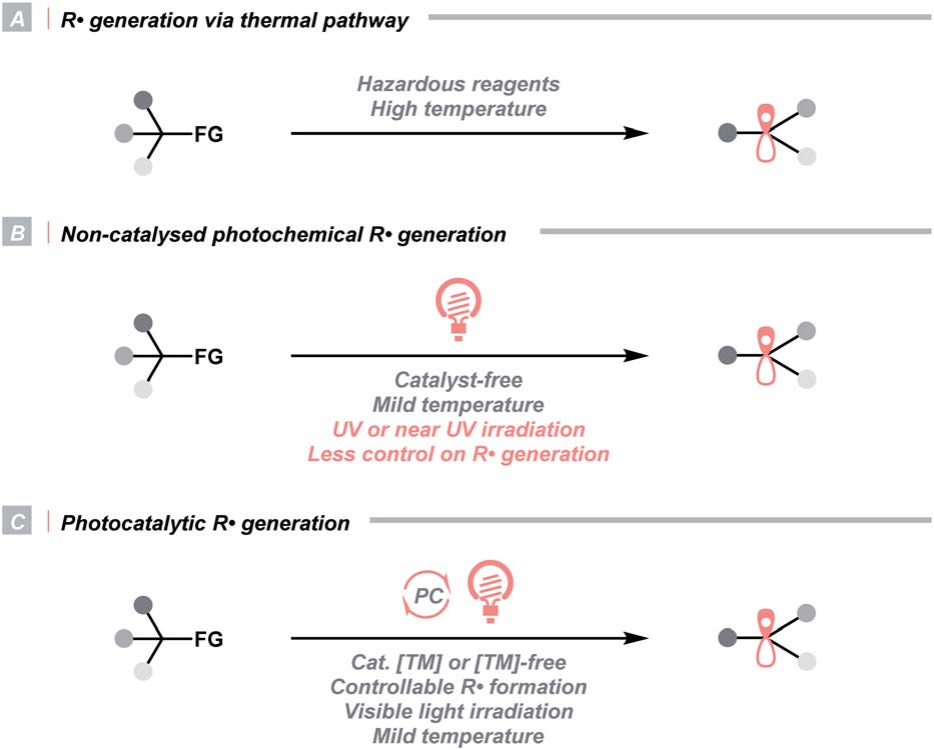
General approaches toward R˙ formation.

Historically, R˙ was rarely involved in reaction designs since it was often produced *via* energy-intensive or user-unfriendly pathways. In these cases, R˙ was less controllable, which could lead to non-productive quenching or other undesired side reactions (Scheme 1A). For instance, thermolysis of peroxides or persulfates at high temperatures exemplified one common practice of delivering R˙ *via* activating aliphatic C–H bonds or carboxyl groups. Utilizing toxic metals such as organotin reagents to fragment alkyl (pseudo)halides was another routine method for R˙ generation. Aside from the thermochemical protocols, direct irradiation of radical precursors in some earlier photochemical strategies could significantly lower the reaction temperature, although ultraviolet (UV) light was often required (Scheme 1B). Consequently, global excitation of reaction components was often inevitable, and radical dimerisation, disproportionation and other off-target processes were frequently observed.

In this context, developing efficient and controllable radical-generating methods for sp^3^ carbon functionalisations was long-sought-after. Pioneered by MacMillan, Stephenson, Yoon and others, photoredox catalysis experienced an impactful renaissance and stood out as a promising solution to previous limitations (Scheme 1C). Unlike conventional approaches, photocatalysis liberates radicals catalytically, usually under visible light, therefore, effectively managing a low concentration of radical species and minimizing the counterproductive radical accumulation. Besides, photocatalysts are commercially available in numerous forms, which could be organic or inorganic, homogeneous or heterogeneous. They are tunable in terms of redox potentials, excited-state energies and other photophysical properties, hence, suitable for multiple synthetic cases. More importantly, the resulting radicals were well orchestrated by the catalysts in these systems, which could elicit reactivities that were unattainable by conventional means, *e.g.*, enantioselective alkylation, radical–radical cross-coupling and photoredox/transition metal dual catalysis.

Armed with these benign features, it is unsurprising that photocatalytic R˙ generation has become the mainstream for radical-based organic synthesis in recent years. Indeed, some of the aforementioned content has been reviewed in the literature, which was categorised either by types of the photocatalysts^1–4^ or bond formation.^5–8^

**Scheme 2:**
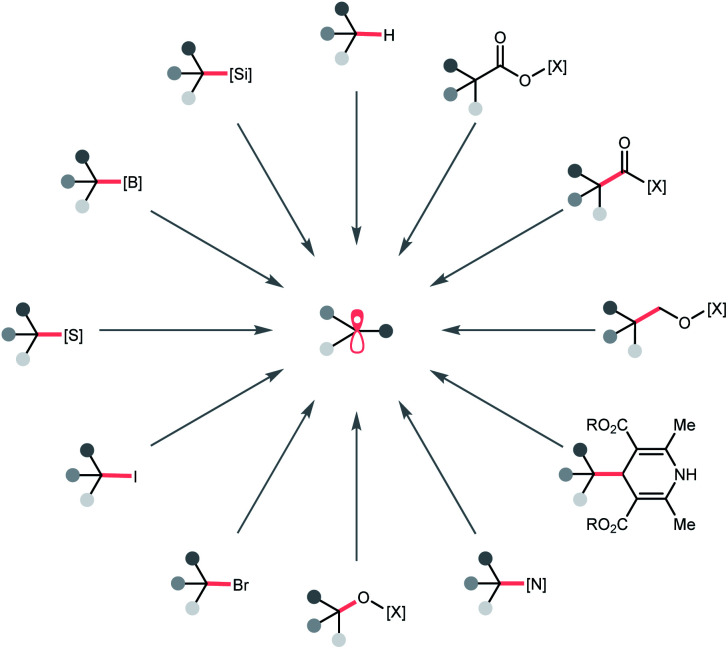
Representative precursors for photocatalytic R˙ formation. [X], activating group.

In this regard, we would like to contribute a review from a different perspective by focusing on the photocatalytic R˙ generation. These elegant reports will be organised based on the key bond cleavage during the R˙ generation, thus, highlighting the diversity of C(sp^3^) radical precursors and the corresponding photocatalytic bond-cleavage strategies and retrosynthetic possibilities (Scheme 2). We envision that this review could provide a quick overview of this field to the audiences, keep them updated with modern pathways to strategise R˙ generation, and facilitate the new design of photocatalysis reactions. Toward this goal, three types of bond cleavages that generate R˙ *via* the cleavage of (a) C–H bonds, (b) C–C bonds, and (c) C–X (X ≠ H and C) bonds will be covered. In the C–H cleavage section, the reactions will be arranged according to hydrogen atom abstractors, while the reactions in C–C and C–X parts will be grouped on a substrate basis.

To be noticed, R˙ in this review referred to all types of C(sp^3^) radicals, including hydrocarbon-based alkyl radicals or C-centred ones that contained at least one heteroatomic substituent (*i.e.*, α-ethereal, α-amino). Giving a comprehensive list of all related literature in this evolving field is beyond our reach; therefore, only representative publications within a decade that featured new mechanistic insights or synthetic applications will be included. To keep this review concise, some photoenzymatic examples from Zhao's and Hyster's groups will not be included.^9–13^

## 2. C–H cleavage

**Scheme 3:**
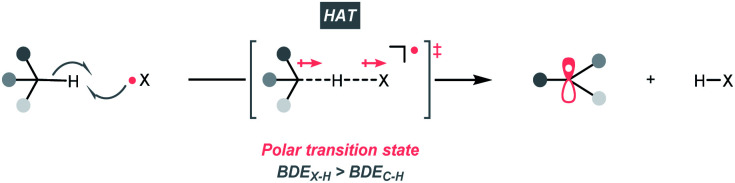
HAT-enabled R˙ generation.

C–H bonds are ubiquitous in organic molecules and readily available in nearly any synthetic stages; therefore, C(sp^3^)–H bonds have been conceived as ideal sources of C(sp^3^) radicals. However, due to their high bond dissociation energies (BDE_C–H_ ∼85 to 105 kcal mol^−1^), the thermodynamic barrier of either direct or indirect hydrogen atom transfer (HAT) with C(sp^3^)–H bonds needs to be overcome.^7,14,15^ Electrophilic hydrogen atom abstractors are often required to cleave C(sp^3^)–H bond homolytically, which are typically electronegative-element-based radicals (X˙) featuring stronger H–X bonds (BDE_X–H_ > BDE_C–H_, Scheme 3). Nonetheless, considering the instabilities of electrophilic radicals and difficulties in distinguishing similar C(sp^3^)–H bonds, enabling the generation of X˙ and selective HAT pose significant challenges in the HAT regime and encourage numerous efforts on designing new HAT reagents and reactions.

Among them, photocatalysis represents one of the state-of-the-art solutions, which could efficiently deliver the electrophilic radicals under mild conditions, set the stage for subsequent HAT and foster the C(sp^3^) radicals. Accordingly, the following sections will be organised based on the types of HAT agents.

### 2.1. HAT with oxy radicals

In light of the high electronegativity of oxygen and the strong O–H bond, oxy radicals should be a potent hydrogen atom abstractor for electron-rich C(sp^3^)–H bonds. Moreover, as oxygenous compounds are abundant, many of them could be used or engineered as oxy radical precursors. Among them, molecular oxygen (O_2_), peroxide and persulfate exemplified some classic options of oxy radical precursors, demonstrating their versatile HAT reactions with C(sp^3^)–H bonds under photocatalytic conditions.^16,17^ Other than these choices, using alkoxides, carboxylates and some inorganic oxides to generate oxy radicals photocatalytically is gaining popularity in recent years.

**Scheme 4:**
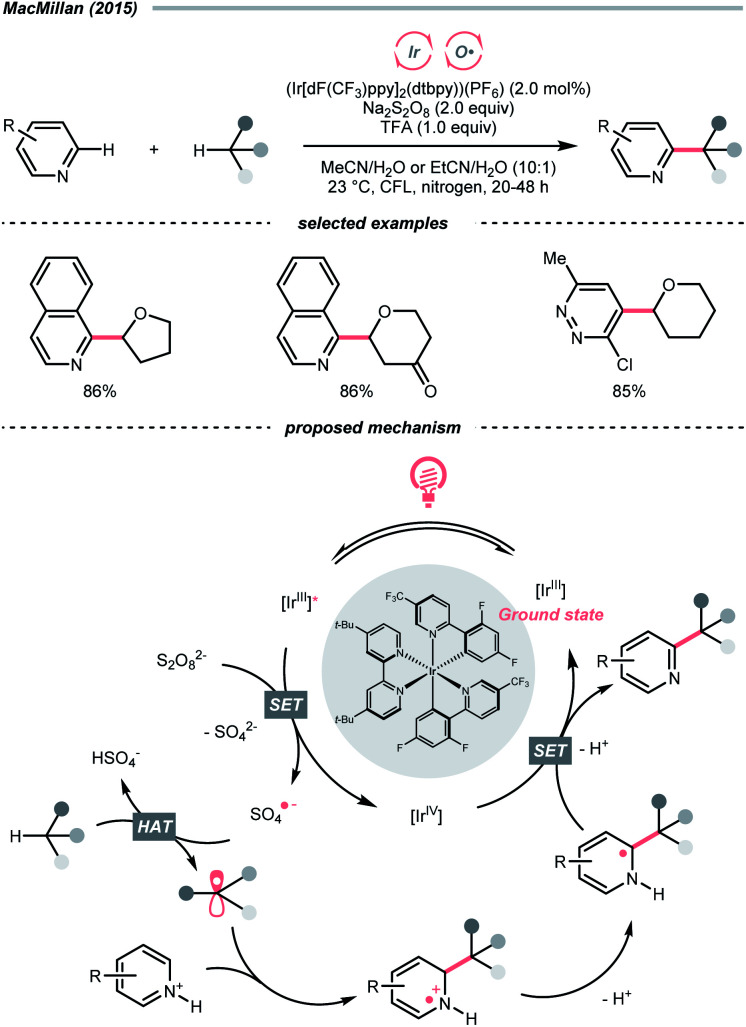
HAT with persulfate.

Mindful of the tunable redox potentials of photoredox catalysts and the weak O–O bonds of peroxides/persulfates, their combination could be a facile method to release oxy radicals catalytically for HAT with C(sp^3^)–H bonds. In 2015, MacMillan's group employed Ir(iii)-photocatalyst and persulfate for Minisci reaction between heteroarenes and ethers (Scheme 4).^18^ Mechanistically, in this photoredox cross-dehydrogenative coupling (CDC),^19–21^ K_2_S_2_O_8_ received an electron from the photoexcited Ir(iii), generating the sulfate radical anion for ethereal α-C–H abstraction. The resulting R˙ was added to the electron-deficient heteroarene, followed by oxidative aromatisation with Ir(iv) to give the desired ethereal heteroarene.

**Scheme 5:**
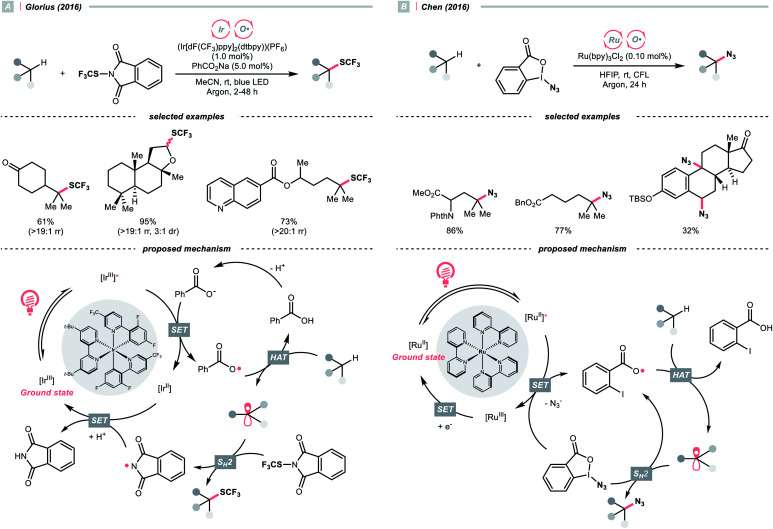
Benzoic acid derivatives as HAT agents.

With the same Ir-photocatalyst, the organic-based benzoate could also serve as the oxy radical precursor. In 2016, the group of Glorius reported site-selective C(sp^3^)–H trifluoromethylthiolations using a catalytic combination of Ir(iii) polypyridyl complex and sodium benzoate under light irradiation (Scheme 5A).^22^ Based on the Stern–Volmer analysis, the benzoate was oxidised by the Ir(iii) to form a benzoyloxy radical (*k*_q_ = 5.6 × 10^7^ M^−1^ s^−1^), which implemented the HAT with R–H to generate an R˙. The higher rate of HAT (*k* = 1.2 × 10^7^ s^−1^) than decarboxylation (*k* = 1.4 × 10^6^ s^−1^) is one crucial concern when choosing the benzoate as co-catalyst. The R˙ then coupled with *N*-trifluoromethylthiolated phthalimide to afford the product. Such an HAT protocol with photocatalytically generated oxy radical exhibited superior site selectivity. Tertiary C(sp^3^)–H was abstracted in preference over secondary and primary ones (>19 : 1 ratio), and the α-oxygenated secondary C(sp^3^)–H was more reactive than the unactivated tertiary ones.

With opposite electronic demand, the aryloxy radical could also be acquired from the reductive SET of the 1,2-benziodoxol-3-(1*H*)-one, a benzoate-type hypervalent iodine. In 2016, Chen *et al.* showcased a C(sp^3^)–H azidation using 1-azido-1,2-benziodoxol-3-(1*H*)-one (BI–N_3_) as both HAT agent and azide source in the presence of a Ru(ii)-photoredox catalyst (Scheme 5B).^23^ Unlike the example in Scheme 5A, single-electron reduction between Ru*(ii) and BI–N_3_ occurred, and the latter underwent O–I cleavage to form the 2-iodobenzoyloxy radical, which was highly selective toward tertiary C(sp^3^)–H bonds. The generated R˙ initiated homolytic substitution with another BI–N_3_ and propagated the radical chain (quantum yield, *ϕ* ∼18). Notably, the authors showed that other than azidation, chlorination and bromination were also achievable by adding corresponding halide salts, which served as halogen sources through the azide-halide exchange with BI–N_3_.

**Scheme 6:**
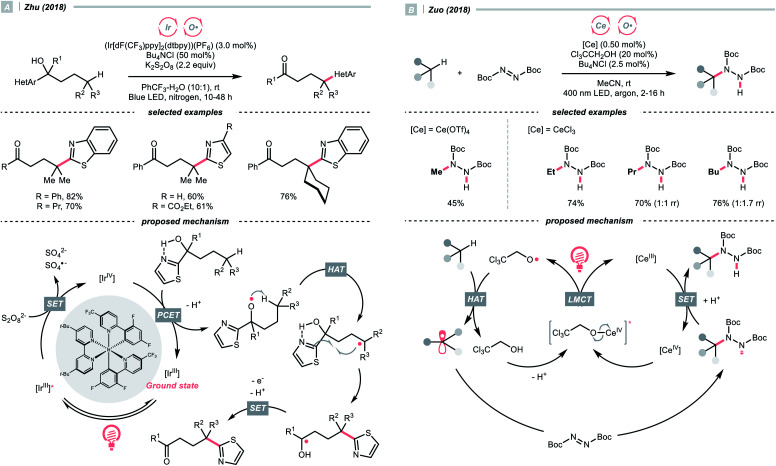
Employing alcohols as HAT agents.

Recently, alcohols were shown as effective organic precursors of oxy radicals, and various strategies were developed to tackle the challenging single-electron oxidation of alcohols. In 2018, Zhu's group documented a photocatalysed remote C(sp^3^)–H heteroarylation reaction (Scheme 6A).^24^ With K_2_S_2_O_8_ as the terminal oxidant, α-heteroaryl tertiary alcohols were converted to γ-heteroaryl ketones through sequential HAT/migratory arylation. An intramolecular proton-coupled electron transfer (PCET) might be operative to facilitate the sluggish oxidation of the –OH group, which was enabled by the cooperative interaction of Ir(iv) species and internal heteroaromatic base. 1,5-Hydrogen atom transfer (1,5-HAT) ensued to give the long-chain R˙ and triggered the heteroarene transfer. The ketyl radical resulted from the migration was then oxidised to the ketone product. In addition to this work, migratory C(sp^3^)–H cyanation, alkenylation and alkynylation were achieved by replacing heteroaromatic moiety under similar photochemical conditions.^25–28^

Ligand-to-metal charge transfer (LMCT) of high-valent metal alkoxide complexes is another powerful means to generate oxy radicals from alcohols, as demonstrated by Zuo and his co-workers (Scheme 6B).^29^ In 2018, they conceived a cerium/alcohol co-catalysed alkane C–H functionalisation reaction and successfully upgraded the light hydrocarbons into value-added products. Albeit the mechanism was still under debate,^30^ Zuo proposed that through the ligand exchange with methanol or 2,2,2-trichloroethanol, the photoreactive cerium alkoxide complex was formed *in situ*, which was excited and homolysed to give the alkoxy radical (RO˙).

Methane, ethane, propane, butane, and cyclohexane were all amenable HAT substrates and functionalised by the di-*tert*-butyl azodicarboxylate (DBAD) to furnish hydrazide products. Such an amination was scalable with continuous-flow reactors. Other than the DBAD, electron-deficient alkene and heteroarene were also applicable alkyl radical acceptors.

**Scheme 7:**
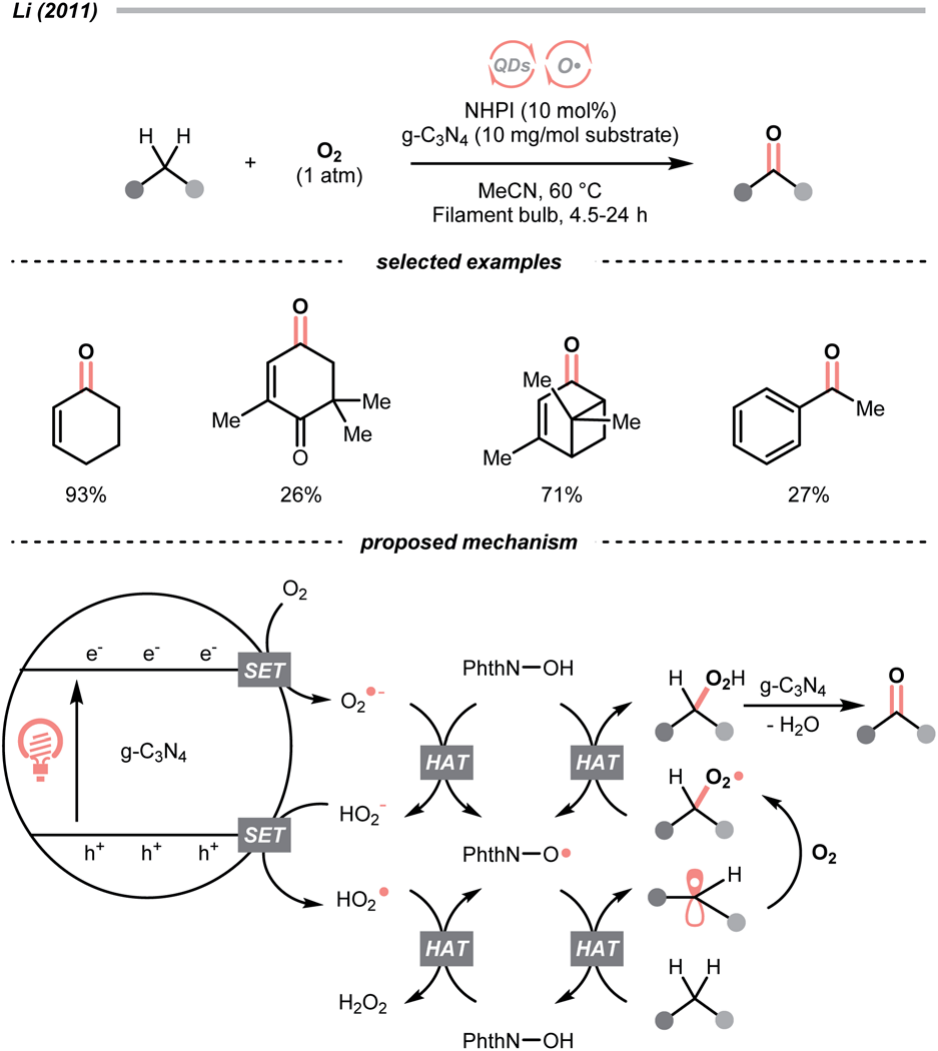
NHPI as HAT agent for C(sp^3^)–H oxygenation.

Similar to alcohols, *N*-hydroxy compounds that could result in *N*-oxy radicals are also viable HAT agents. In 2011, Li and co-workers exploited *N*-hydroxyphthalimide (NHPI) for C(sp^3^)–H oxygenation with graphitic carbon nitride (g-C_3_N_4_) under photoirradiation (Scheme 7).^31^ In the presence of g-C_3_N_4_ catalysts, O_2_ mediated the *N*-oxy radical generation from NHPI, which was proposed as the key radical species to transform allylic or benzylic C–H bonds into C=O. Likewise, Gong *et al.* utilised *N*-hydroxysuccinimide (NHS) as the HAT agent for formylation of fluoroalkyl imines through the HAT of 1,3-dioxolane, wherein diacetyl was responsible for the oxidation of NHS to generate the *N*-oxy radical.^32^

Interestingly, some common inorganic salts could produce oxy radicals, however, under strongly oxidizing conditions. As such, these classes of oxy radical precursors remained underutilised thus far. Organophotoredox catalysts (OPCs) often possess broad redox windows, thereby representing ideal chaperones of the inorganic oxides to give oxy radicals under visible light irradiation.

**Scheme 8:**
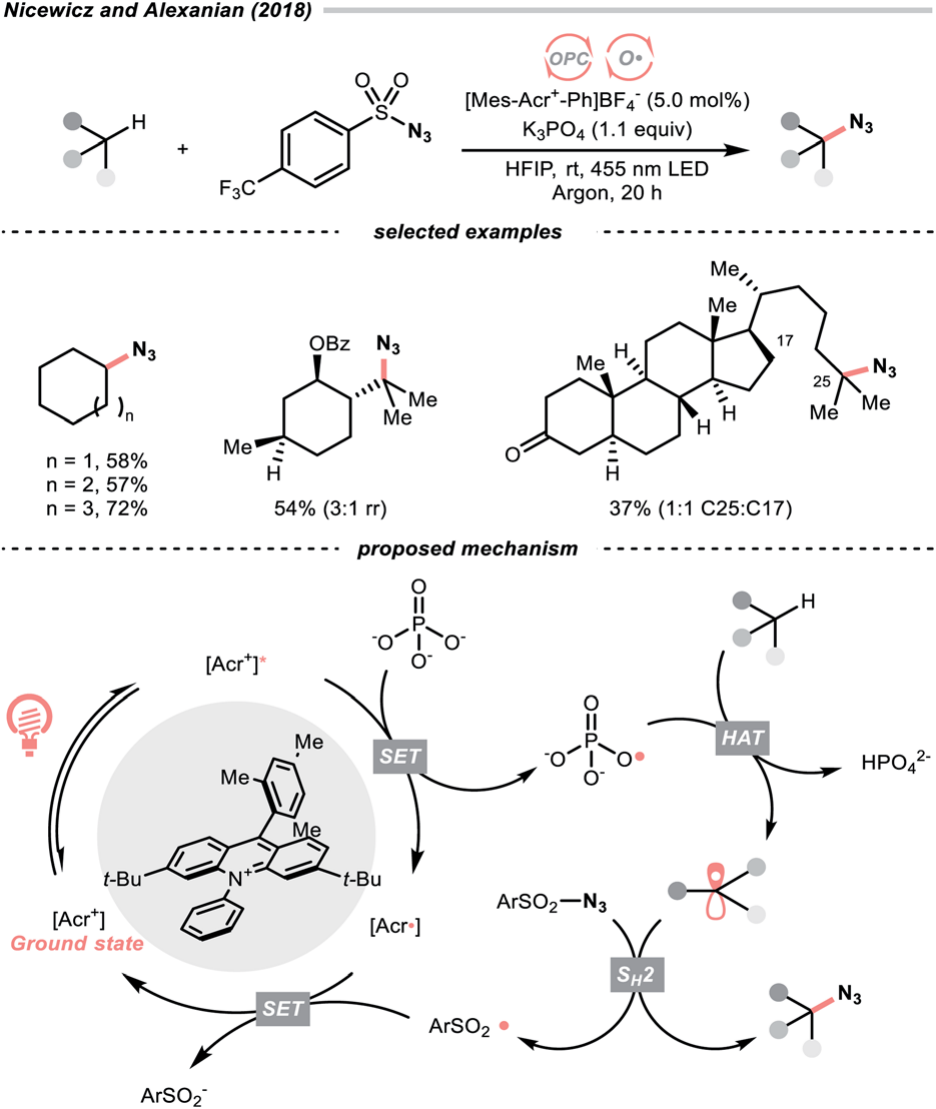
Phosphate as HAT agent.

In 2018, the collaboration between Nicewicz's and Alexanian's group showed that the strongly oxidising excited acridinium OPC could catalyse the azidation of non-activated C(sp^3^)–H bond using K_3_PO_4_ as the HAT agent (Scheme 8).^33^ Taking advantage of the photoexcited acridinium [Mes-Acr^+^-Ph]* (Nicewicz's catalyst, 
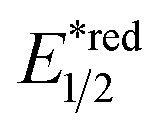
 = +2.08 V *vs.* SCE in MeCN), K_3_PO_4_ was directly turned into the phosphate radical and afforded the R˙ *via* HAT with R–H. Aside from azidation, platform reactions, including fluorination, chlorination, bromination, trifluoromethylation, and alkylation, were successful with the corresponding functionalising reagents to trap the R˙.

**Scheme 9:**
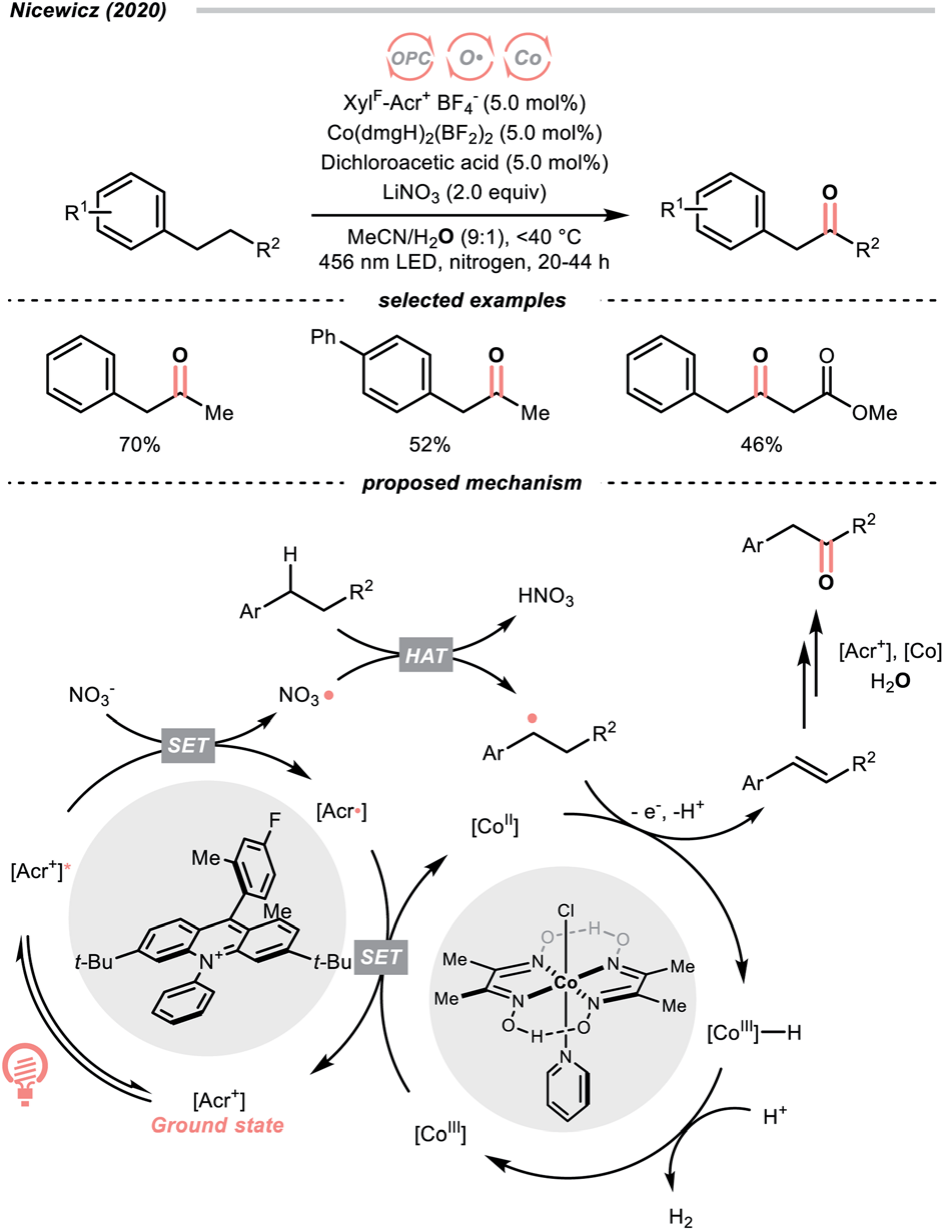
Employing nitrate as HAT agents.

Another inorganic oxide, nitrate, has also been employed for HAT by Nicewicz's group with a similar acridinium OPC (Scheme 9).^34^ In general, benzylic C–H bonds are more prone to oxidation relative to their adjacent analogues due in part to the weaker bonding of the former. In Nicewicz's homobenzylic oxygenation reaction of alkylarenes, an OPC/Co dual catalytic system was employed to tackle the challenging C–H oxygenation at homobenzylic positions in favour of benzylic ones. In the plausible mechanism, the nitrate was first oxidised by the photoexcited acridinium, generating a nitrate radical for benzylic HAT. The benzylic radical (R˙) was intercepted by the cobaloxime catalyst to yield a styrene intermediate, which was further subjected into anti-Markovnikov Wacker-type olefin hydration, granting the benzyl ketone product. Interestingly, homobenzylic oxidation could still occur without nitrate for electron-rich substrates.

**Scheme 10:**
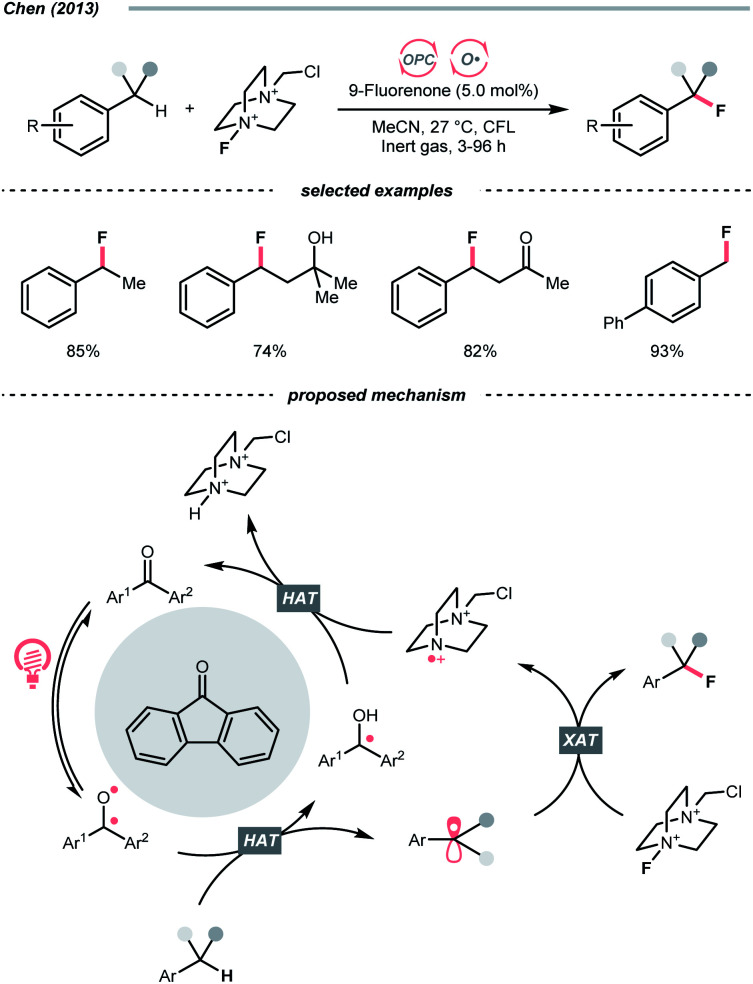
Ketone-catalysed C(sp^3^)–H fluorination.

Complementary to the examples above, which accommodated separate photocatalyst and oxy radical precursor in their conditions, some photocatalysts themselves could behave as oxy radicals after light excitation. Ketone is a well-known representative in this class, considering the long and prosperous history of Norrish chemistry. In 2013, Chen's group reported a photocatalytic benzylic C–H fluorination using 9-fluorenone as the HAT agent with Selectfluor as the fluorine source (Scheme 10).^35^ Under compact fluorescent lamp (CFL) irradiation, the triplet diarylketone abstracted hydrogen at the benzylic position and generated the benzyl radical (R˙). The Selectfluor served as both the fluorine atom source to fluorinate the R˙ and the oxidant for photocatalyst turnover. Interestingly, switching to xanthone gave divergent reaction outcomes under the same photo conditions, making benzyl difluorides the major products. Moreover, this photocatalytic C(sp^3^)–H fluorination was also feasible for cyclic and acyclic alkanes.

**Scheme 11:**
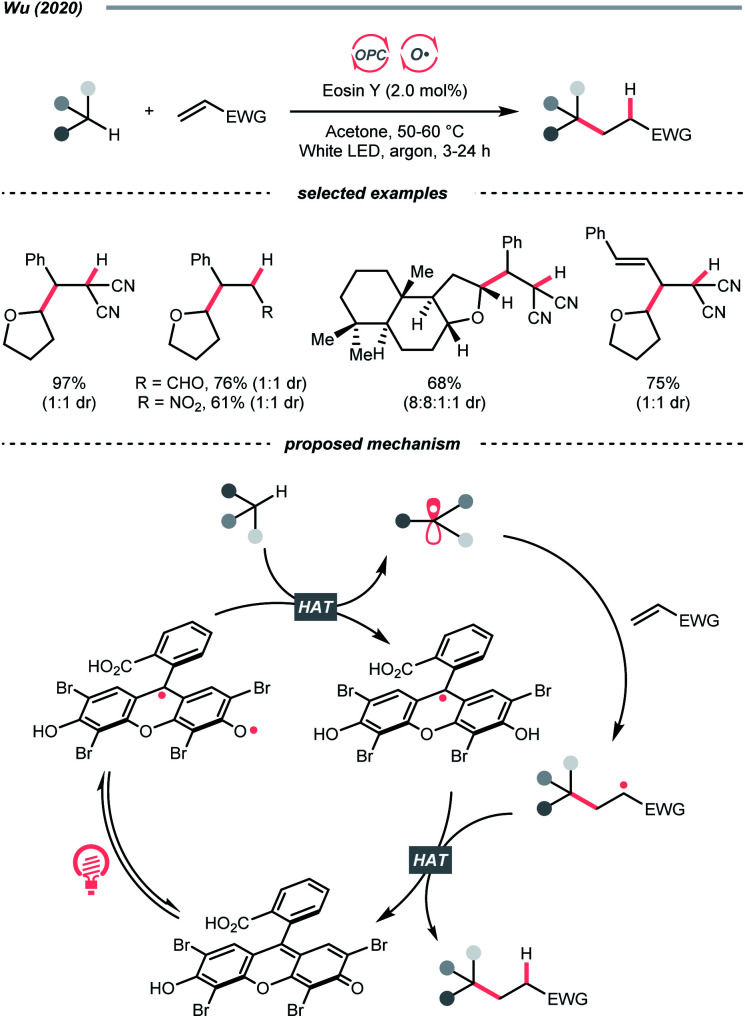
Eosin Y-catalysed C(sp^3^) radical generation.

Organic dye eosin Y also belongs to this class of photosensitiser. It is visible-light-absorbing and normally used for the single-electron transfer (SET) and energy transfer (EnT) catalysis. In 2020, Wu's group discovered a neutral-eosin Y-catalysed Giese reaction between the ethers and alkenes, in which the underexplored HAT capability of eosin Y was revealed (Scheme 11).^36^ Mechanistically, the neutral eosin Y, more specifically, its *para*-quinone methide moiety, was excited to a diradical form and responsible for the HAT with ethereal α-C–H bond. Then, the R˙ was engaged in the Giese reaction with electron-deficient double bonds to give the R′˙.

Owing to the steric and captodative effect, the reduced eosin Y radical intermediate was relatively stable, which was reformed into the active catalyst by another formal HAT with the R′˙. As such, good to excellent yields of Giese reaction products were also obtained with other C(sp^3^)–H substrates like alcohols, and amides, while cyclohexane gave only a poor yield. It was worth mentioning that moderate heating was required to increase the reaction efficiency, and eosin Y disodium salt (Na_2_-eosin Y) was ineffective for this chemistry.

**Scheme 12:**
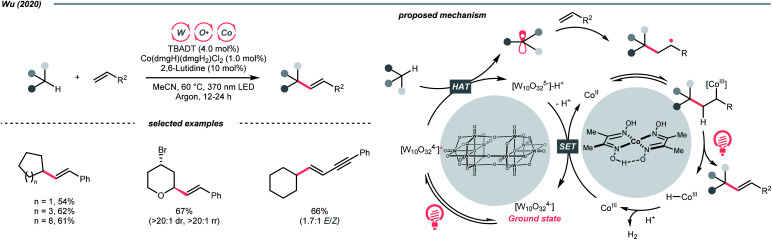
TBADT-catalysed C–H alkenylation of alkanes.

Beyond organophotocatalysis, some metal oxides such as decatungstate (W_10_O_32_^4−^, DT), uranyl dication (UO_2_^2+^),^37^ and antimony porphyrin complexes (SbTPP)^38^ could host formal oxy radicals on their periphery under near-ultraviolet (UVA) light irradiation. Capitalizing on this property, Wu *et al.* designed a CDC reaction^19–21^ between alkanes and alkenes by merging tungsten and cobalt catalysis (Scheme 12).^39^ In this dual catalysis system, tungsten was responsible for R˙ generation *via* HAT, while cobalt was proposed to turn over such a net oxidative coupling *via* H_2_ evolution. Specifically, HAT occurred between the photoexcited decatungstate [W_10_O_32_^4−^]* and alkane, affording an R˙ and [W_10_O_32_]^5−^H^+^. While R˙ underwent the Giese addition toward alkenyl C=C bond and produced a new stabilised R′˙, the latter (*E*^red^_1/2_ = −0.97 V *vs.* SCE in MeCN) reduced the Co(iii) (*E*^red^_1/2_ = −0.16 V *vs.* SCE in MeCN) into Co(ii). Binding of Co(ii) and R′˙ followed by β-hydride elimination furnished the alkylated alkene products with a high *E*/*Z* ratio and a Co(iii)–H. Later, the cobaloxime cycle was closed by quenching the Co(iii)–H with proton and releasing H_2_.

### 2.2. HAT with nitrogen-centred radicals

Adjacent to oxygen, nitrogen is also a highly electronegative element, and its radicals are suitable for the HAT with C(sp^3^)–H bonds. Early examples of N-centred radical (NCR) could be traced back to the Hofmann–Löffler–Freytag (HLF) reaction, an intramolecular NCR-mediated HAT reaction.^40,41^ Although nitrogen is less electronegative than oxygen, NCRs could fine-tune their steric and electronic properties by varying the *N*-substituents.

**Scheme 13:**
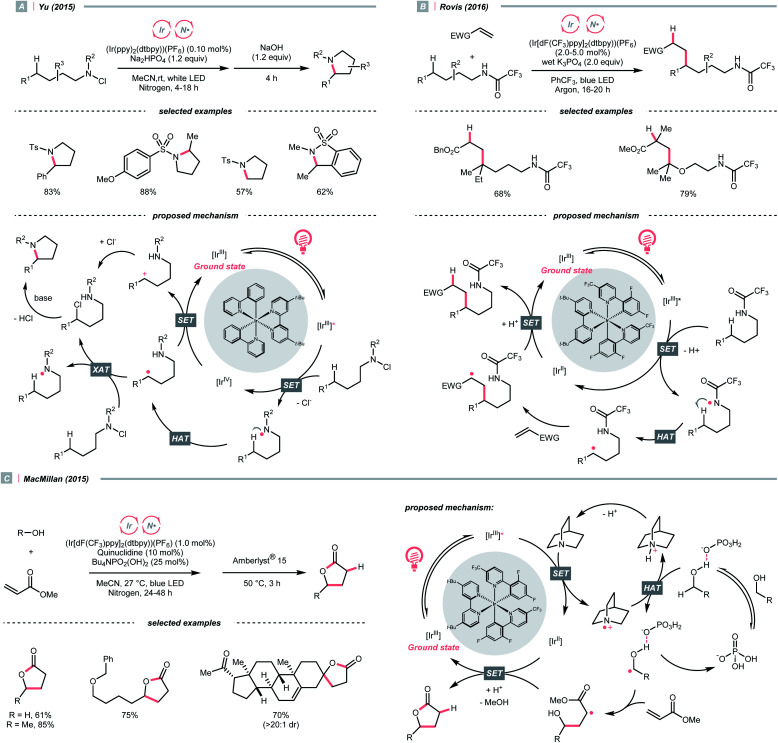
NCR-assisted remote C–H functionalisations.

A photocatalytic example of an HLF reaction to synthesise cyclic amides from *N*-chloroamides was developed by Yu's group in 2015 (Scheme 13A).^42^ The photoreduction of the *N*-chloroalkyl amide by Ir*(iii) induced the N–Cl cleavage and formed an NCR, which underwent 1,5-HAT with its pendant alkyl chain to engender a C(sp^3^) radical (R˙). The R˙ could be oxidised by Ir(iv) following chloride attack, or alternatively, chlorinated by another *N*-chloroamide, to afford a 5-chloroalkyl amide. With the stepwise addition of a base, intramolecular S_N_2 amidation occurred, giving the cyclic amide products.

A similar photocyclisation reaction could also be realised using simple secondary amides as NCR precursors as well as HLF substates, which was revealed by Rovis *et al.* in 2016 (Scheme 13B).^43^ Based on their mechanistic studies, acidic amide substrates were crucial, which was partially deprotonated and subjected to oxidation with Ir*(iii). Alternatively, PCET might be operative. The NCR triggered the C(sp^3^) radical generation *via* 1,5-HAT, which was engaged in the Giese reaction with electron-deficient olefins.

Concurrently, the group of Knowles reported the same photocatalytic remote C–H alkylation but with catalytic Bu_4_NPO_2_(OBu)_2_ as the base.^44^ Inspired by these elegant chemistries, several amide catalysts were designed to pair with the Ir-photocatalyst, enabling a series of C(sp^3^)–H alkylation,^45^ alkenylation^46^ and arylation.^47^

In addition to amides and their derivatives, simple amine could accomplish the HAT/Giese reaction sequence under photocatalytic conditions. In 2015, MacMillan *et al.* reported an iridium/quinuclidine/phosphate triple-catalysed photoredox reaction between alcoholic α-C(sp^3^)–H bonds and electron-poor alkenes (Scheme 13C).^48^ In their tentative mechanism, the NCRs was generated from the oxidation of quinuclidine (*E*^red^_1/2_ = +1.10 V *vs.* SCE in MeCN) by the photoexcited (Ir[dF(CF_3_)ppy]_2_(dtbpy))(PF_6_) (*E*^red^_1/2_ Ir*(iii)/Ir(ii) = +1.21 V *vs.* SCE in MeCN), producing the key R˙ selectively at the alcoholic α-position. The unique regioselectivity, in this case, might stem from hydrogen bonding between alcoholic O–H and phosphate, which weakened the α-C(sp^3^)–H of alcohol. Such an interaction would allow the selective alkylation with strong C(sp^3^)–H bond in the presence of weaker ones such as allylic, benzylic, α-ethereal and α-carbonyl C(sp^3^)–H bonds.

**Scheme 14:**
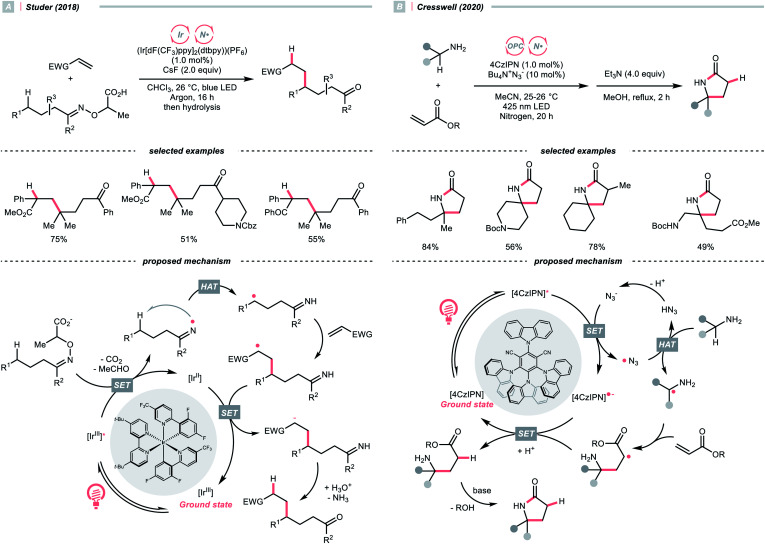
Amine- and azide-catalysed α-C(sp^3^)–H functionalisations.

Apart from amines and amides, which formed sp^3^-hybridised NCRs, sp^2^ nitrogen radicals are also effective in HAT. Among them, iminyl radicals were often employed in imine remote C(sp^3^)–H functionalisations, giving ketones after hydrolysis.^49,50^ Taking Studer's γ-alkylation of ketone as an example (Scheme 14A),^51^ an α-aminoxy acid auxiliary was condensed with the ketone, of which the carboxylate group could be oxidised by the Ir*(iii) to implement decarboxylation and deacetylation, giving an iminyl radical for remote C(sp^3^)–H abstraction.

In 2020, Cresswell and his team conceived an α-tertiary amine synthesis *via* azide radical-enabled HAT with unmasked amines (Scheme 14B)^52^ Similar to MacMillan's quinuclidine radical-mediated HAT (Scheme 13C), the anionic azide was oxidised by photoexcited 1,2,3,5-tetrakis(carbazol-9-yl)-4,6-dicyanobenzene (4CzIPN) to provide an azide radical 
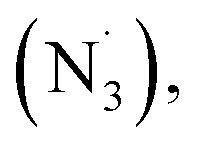
 which performed regioselective C(sp^3^)–H abstraction from an α-secondary amine and delivered an α-amino R˙. After R˙ addition to a conjugate alkene, the resulting amine could be isolated or subsequently cyclised to afford a lactam.

**Scheme 15:**
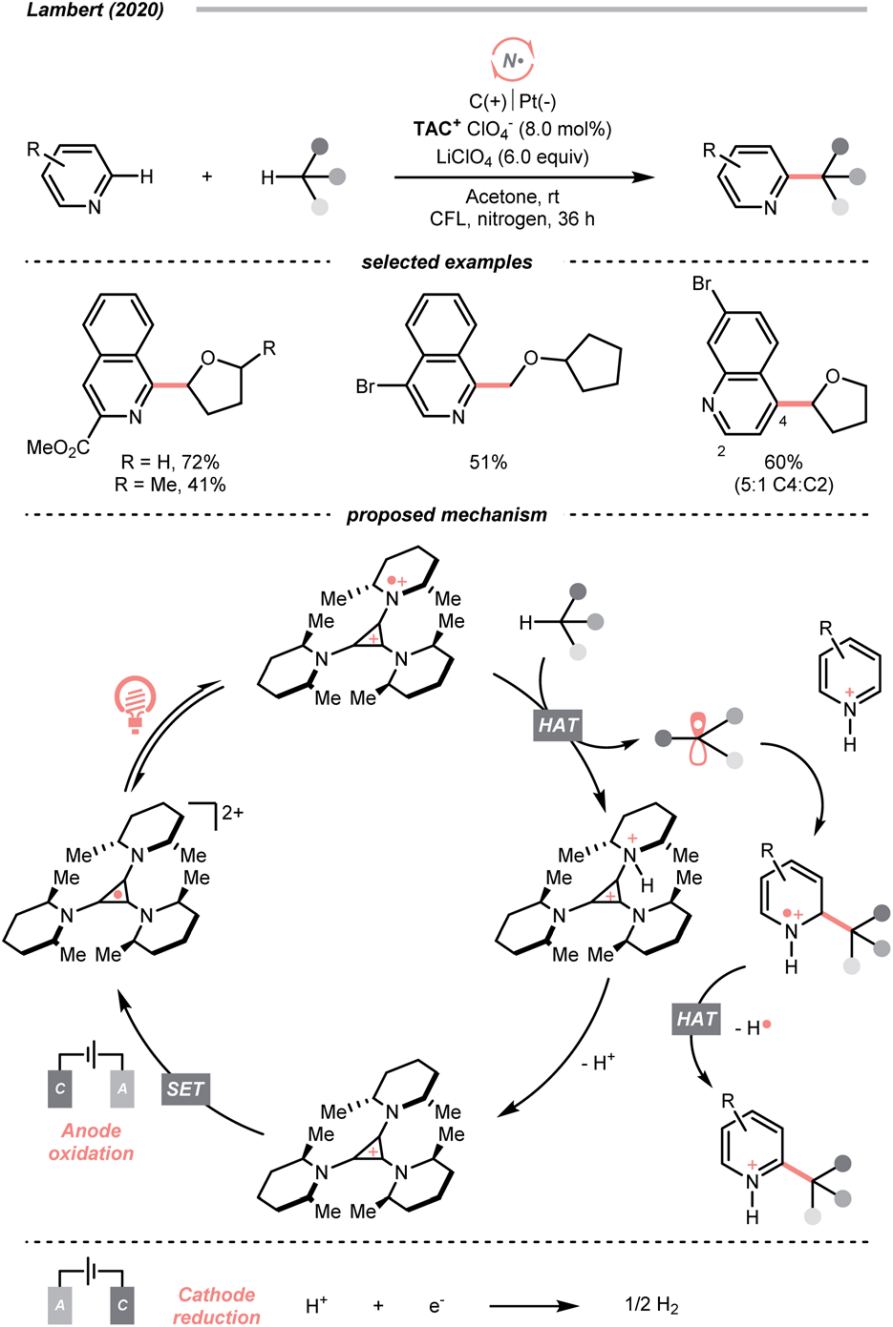
TAC-catalysed HAT under photoelectrochemistry conditions.

Similar to oxy radicals, some special photocatalysts could serve as NCR precursors directly, albeit rarely reported in the literature. For instance, trisaminocyclopropenium ion (TAC), which Lambert's group widely applied, was reported to enable site-selective heteroarylation of ether under photoelectrochemical conditions (Scheme 15).^53^ According to their proposed mechanistic rationale, the TAC was first oxidised into TAC˙^+^*via* anodic oxidation. After photoexcitation, it was transformed into an aminyl radical cation, which could abstract the ethereal α-C(sp^3^)–H to produce R˙ or rearomatise R˙/heteroarene adduct to give the Minisci reaction products. On the other side, cathodic reduction of H^+^ would release H_2_. Following the success of this work, C(sp^3^)–H diamination and amidation were later achieved using the same TAC photocatalyst.^54,55^

### 2.3. HAT with thiyl radicals

Moving downward in the chalcogen column, S-centred (thiyl) radicals, despite being less electrophilic relative to oxy radicals, could also perform HAT with some C(sp^3^)–H bonds. In general, thiyl radicals could be generated more easily because of the more polarisable and less electronegative sulfur centres.

**Scheme 16:**
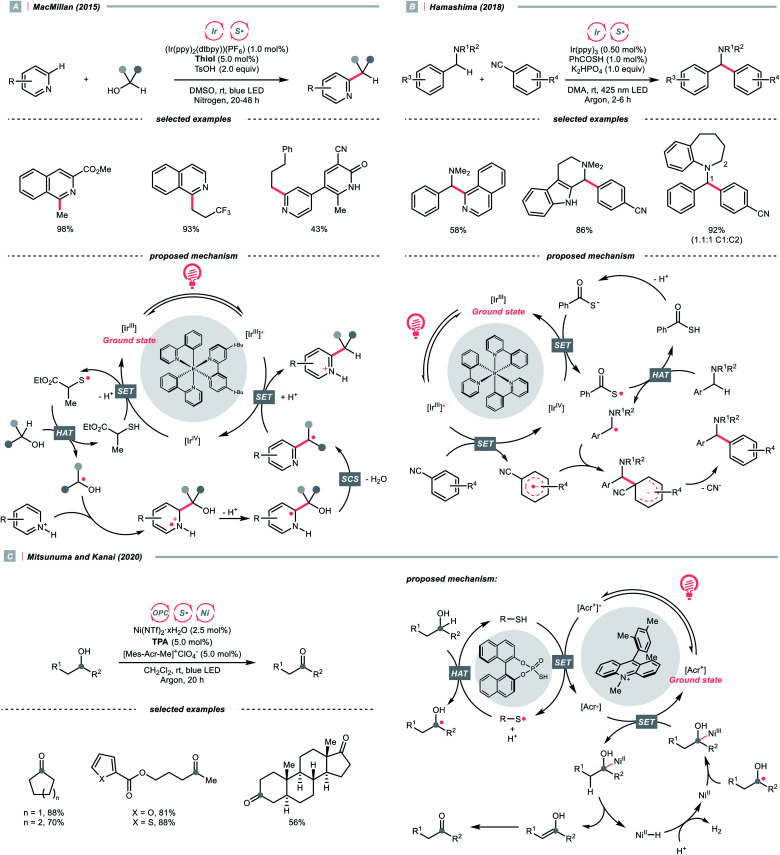
Thiyl radical-enabled C(sp^3^)–H elaborations.

Like the oxy radical, thiyl radicals can be formed from thiols, thiocarboxylic acids and thiophosphoric acids.^56,57^ By merging a thiol catalyst and Ir-photocatalyst, the group of MacMillan reported a dehydrative Minisci alkylation using thiol as the HAT agent and alcohols as the alkyl sources (Scheme 16A).^56^ Mechanistically, the essential thiyl radical came from the SET between [Ir(ppy)_2_(dtbpy)_2_]^2+^ (*E*^red^_1/2_ Ir(v)/Ir(iii) = +1.21 V *vs.* SCE in MeCN) and mercaptan co-catalyst (*E*^red^_1/2_ = +0.85 V *vs.* SCE in MeCN for cysteine). Then, the thiyl radical abstracted the hydrogen atom from alcoholic α-C(sp^3^)–H assisted by the polar effect; otherwise, such transformation would be thermodynamically unfavourable (BDE_S–H_ for thiol ∼87 kcal mol^−1^; BDE_C–H_ for MeOH = 96 kcal mol^−1^). The nucleophilic addition of R˙ to the protonated heteroarene, which was followed by a spin-centre shift (SCS)-induced dehydration and some proton/electron transfer steps to give the alkylated Minisci products. Notably, the successful application of methanol for aromatic methylation represented a major breakthrough in the field.

Using thiobenzoate as a catalytic HAT agent, Hamashima *et al.* reported a redox-neutral C(sp^3^)–C(sp^2^) coupling of benzyl amines and cyanoarenes in aid of Ir-photocatalyst (Scheme 16B).^58^ The photocatalytic cycle was initiated by reduction of the photoexcited Ir(iii) by dicyanoarene to form a radical anion intermediate. Subsequently, electron transfer between Ir(iv) and thiobenzoate oxidised the latter and generated the thiyl radical for benzylic HAT. Due to the persistent radical effect, coupling the α-aminobenzylic radical (R˙) and cyanoaryl radical anion was feasible, which could afford the product after cyanide extrusion.

Switching to another thiyl radical HAT system with acridinium, thiophosphoric acid (TPA) and Ni(ii), Mitsunuma and Kanai reported a photocatalytic acceptorless dehydrogenation reaction of alcohols in 2020 (Scheme 16C).^59^ Based on their seminal results,^60^ a triple catalytic cycle was designed. The TPA was oxidised by the excited Fukuzumi catalyst to form a thiyl radical for HAT with the alcoholic α-C–H bond. The ensuing R˙ was intercepted by nickel(ii), followed by β-hydride elimination and tautomerisation to deliver the ketone product. Terminal oxidant was absent in this reaction because the nickel promoted the H_2_ evolution and closed the catalytic cycle. Notably, under the same C(sp^3^) radical generation scenario, intermolecular reactions such as the Giese reaction and oxidative esterification between alcohols and aldehydes were successful.

### 2.4. HAT with halogen radicals

The application of halogen radicals in organic synthesis could date back to more than 150 years ago when Regnault discovered that dichloromethane could be formed by exposing chloromethane and chloroform to sunlight.^61^ While the Cl˙-involved process remains a common practice for alkyl chloride synthesis, many novel C(sp^3^)–H functionalisation reactions have been established by embedding the halogen radical-mediated HAT in visible light photocatalysis.

**Scheme 17:**
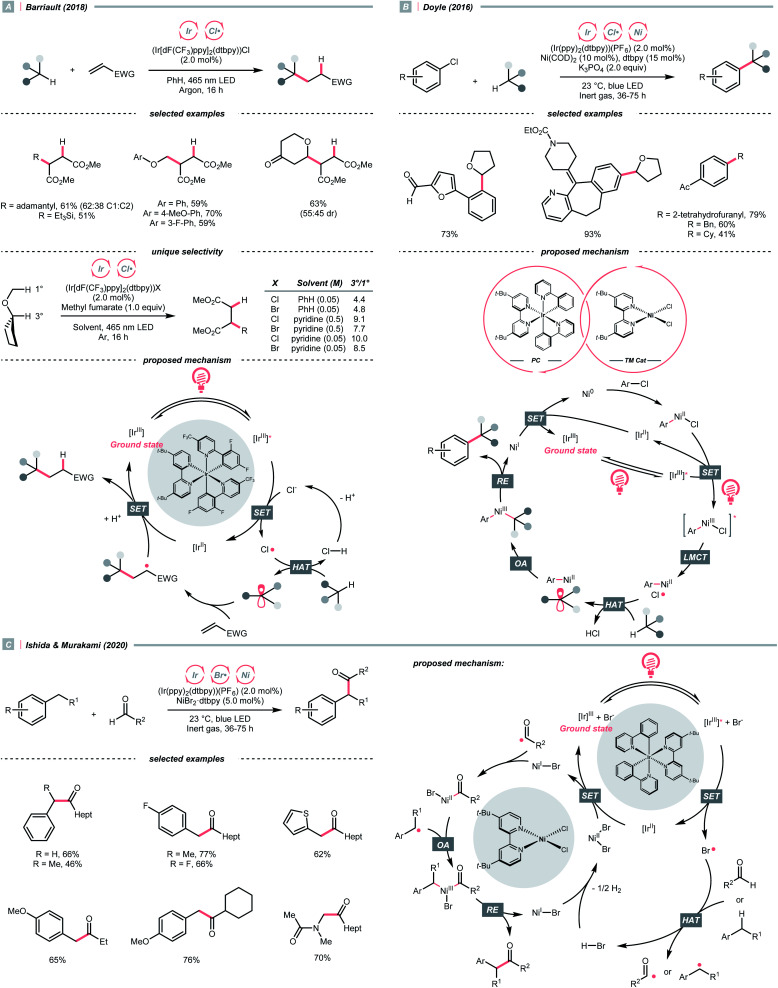
Halogen radical-enabled C(sp^3^)–H activations.

Inorganic chlorides (Cl^−^) represent a convenient source of Cl˙ for laboratory synthesis. However, oxidation of Cl^−^ to Cl˙ (*E*^red^_1/2_ = +2.03 V *vs.* SCE in MeCN) mandates strong oxidants,^62^ and controlling the reactivity of Cl˙ stays challenging. In 2018, Barriault and his group reported an elegant solution to solve these two problems and realised an (Ir[dF(CF_3_)ppy]_2_(dtbpy))Cl-catalysed Giese reaction with alkanes (Scheme 17A).^63^ Mechanistically, a radical process with Cl˙ and R˙ was proposed. The former was produced from the SET between excited Ir(iii) and chloride under gentle heating conditions since the Cl^−^ oxidation was unfavourable in this case (*E*^red^_1/2_ Ir*(iii)/Ir(ii) = +1.21 V *vs.* SCE in MeCN). The latter was derived from the HAT between Cl˙ and alkanes and was subjected to the Giese pathway. Interestingly, the reactivity of Cl˙ could be tamed at low concentration with pyridine as the solvent, wherein it exhibited enhanced selectivity toward tertiary C(sp^3^)–H bonds than others in cyclopentyl methyl ether.

Complementary to SET, LMCT is also effective in generating Cl˙.^62,64^ Inspired by Nocera's observation on photoinduced LMCT of nickel(iii) trichloride,^65^ in 2016, Doyle and colleagues designed a dual metallaphotoredox catalysis reaction for a redox-neutral coupling of aryl chlorides and ethers (Scheme 17B).^62^ By merging iridium photocatalysis and nickel catalysis under visible light, the key Cl˙ could result from an LMCT process of the excited Ni(iii)(Ni–Cl. This strategy could bypass the unfavourable outer-sphere oxidation of Cl^−^ by photocatalyst, and external Cl^−^ was absent in Doyle's conditions since oxidative addition of Ni(0) could gain Cl^−^ from a broad range of aryl chlorides. Noticeably, C(sp^3^)–H arylations with toluene and cyclohexane were also feasible, albeit in lower yields.

Consistent with the trend of the oxygen-to-sulfur switch, compared to Cl^−^, bromide (Br^−^) has a less positive reduction potential (*E*^red^_1/2_ = +1.60 V *vs.* SCE in MeCN), weaker hydrogen–halide bond (BDE for H–Br = 87 kcal mol^−1^) and lower electronegativity; therefore, bromine radical (Br˙) could be a theoretically more selective HAT agent that is easier to obtain.^66^ Based on these properties, Ishida and Murakami *et al.* utilised the nickel/iridium dual metallaphotocatalytic system for the CDC between toluene derivatives and benzaldehydes (Scheme 17C).^67^ The direct Br^−^-to-Ir*(iii) electron transfer of the *in situ* formed [Ir(ppy)_2_(dtbpy)]Br led to the Br˙ formation. Impressively, the yield of cross-coupling products could be optimised by fine-tuning the molar ratio of toluenes and aldehydes.

As demonstrated by Wu's laboratory, the same Br˙ could also be derived from the CH_2_Br_2_ oxidation by photoexcited acridinium catalyst, which was submitted to achieve alkyl C–H abstraction for Giese reaction.^68^ To be noted, the HAT byproduct, HBr, could serve as additional Br˙ sources.

### 2.5. HAT with carbon-centred radicals

Unlike heteroatom-based radicals, most non-functionalised C-centred radicals are nucleophilic. Since the components on both sides of the HAT equation are very similar in terms of the C–H bond strength and C-centred radical polarity, low kinetics of the HAT step, premature coupling process and other side reactions are major concerns of this HAT protocol. This explained its rare application in the intermolecular process.

However, C-centred radical-mediated HAT enjoyed rapid development in recent years owing to the renaissance of photocatalysis. Along this line, Gevorgyan, Reiser, Zhu and other research groups have devoted themselves to advancing HAT chemistry with C-centred hydrogen abstractors in transition metal-catalysed or metal-free reactions.^69–71^

**Scheme 18:**
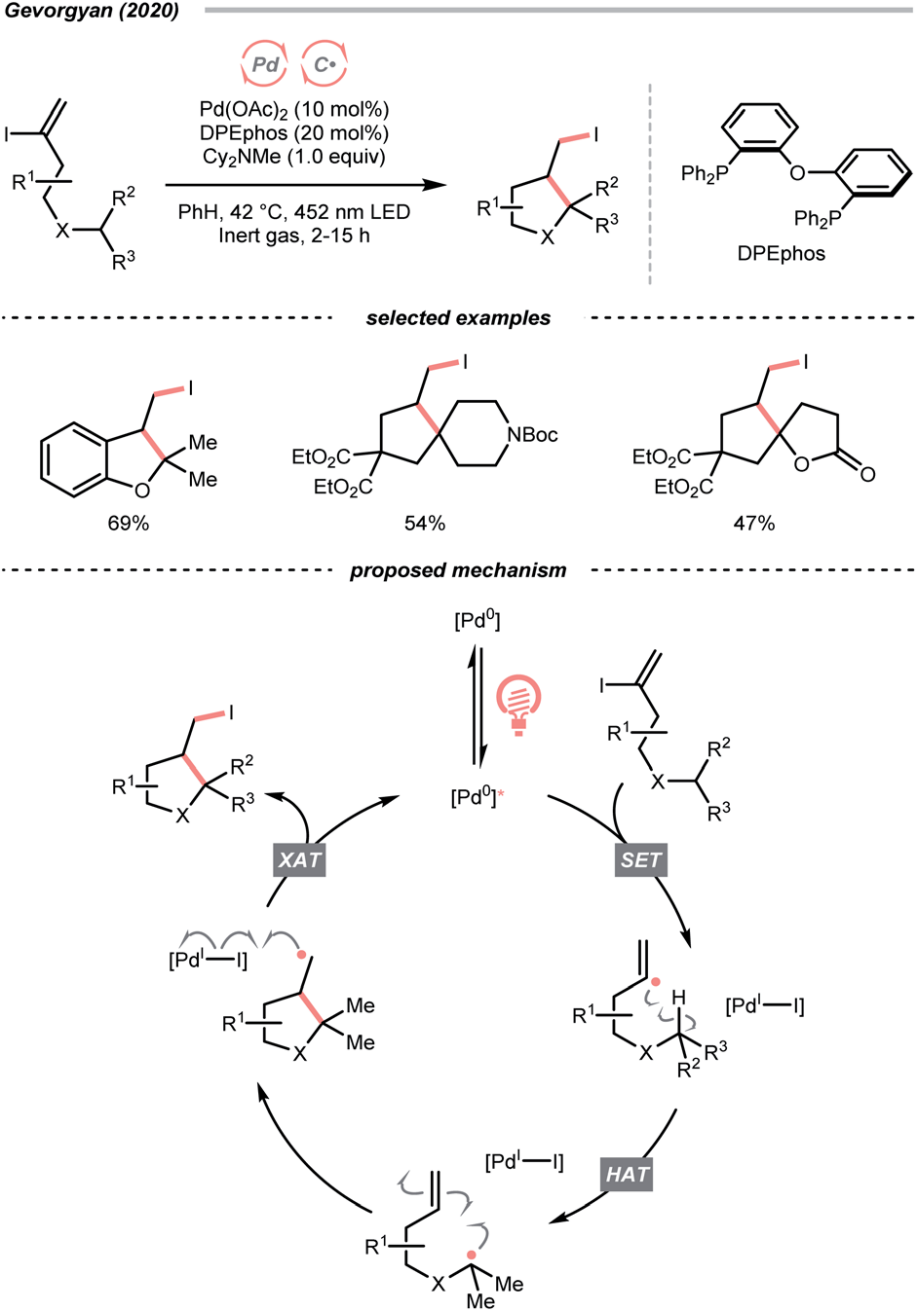
Vinyl radical as HAT agent.

In 2020, Gevorgyan's group described a photoinduced intramolecular atom-transfer radical cyclisation (ATRC) reaction of vinyl iodides to synthesise 3-iodomethyl dihydrobenzofurans under palladium photocatalysis (Scheme 18).^72^ An unprecedented hybrid vinyl/Pd(i) radical pair intermediate was proposed as a consequence of SET between the photoexcited Pd(0) catalyst and vinyl iodide. A 1,5-HAT process between the vinyl radical and tertiary C(sp^3^)–H bonds then proceeded, generating an R˙ for the iodocyclisation.

**Scheme 19:**
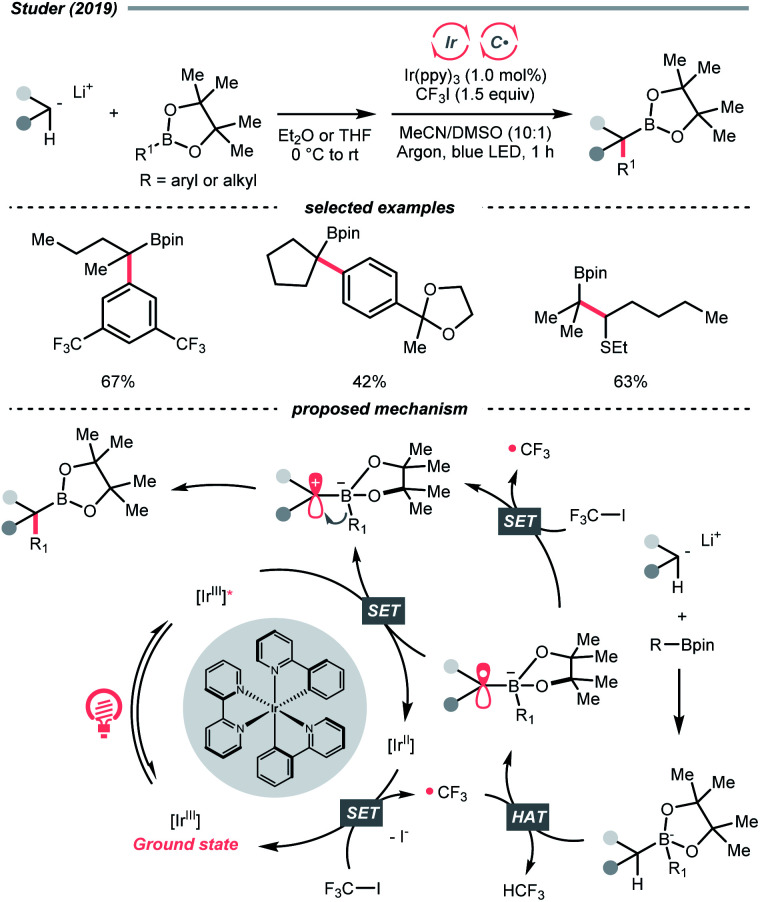
Trifluoromethyl radical-mediated C(sp^3^)–H arylation.

Other than C(sp^2^)-centred radicals, C(sp^3^)-centred radicals were also versatile HAT agents. In 2019, Studer's group reported photocatalysed α-C–H alkylation and arylation of alkylboronic esters, in which trifluoromethyl iodide mediated the HAT under photocatalytic conditions (Scheme 19).^73^ In their original mechanistic proposal, the trifluoromethyl radical 
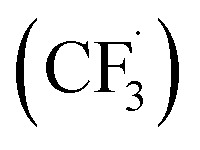
 was generated through SET between the CF_3_I (*E*^red^_1/2_ = −1.52 V *vs.* SCE in DMF) and Ir*(iii) (*E*^red^_1/2_ Ir(iv)/Ir*(iii) = −1.73 V *vs.* SCE in MeCN). The 
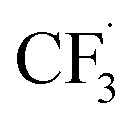
 could execute HAT with the pre-synthesised boronate complex and form a radical anion intermediate (R˙), which was further oxidised by Ir(iv) or another trifluoromethyl iodide and underwent 1,2-alkyl or aryl migration to afford the α-substituted boronates. It should be noted that the radical chain process was supported by the reaction quantum yield measurement (*ϕ* = 8.8). Also, the fluorescence quenching of the Ir-photocatalyst with the boronate complex indicated another plausible catalytic cycle initiated by the borate oxidation.

**Scheme 20:**
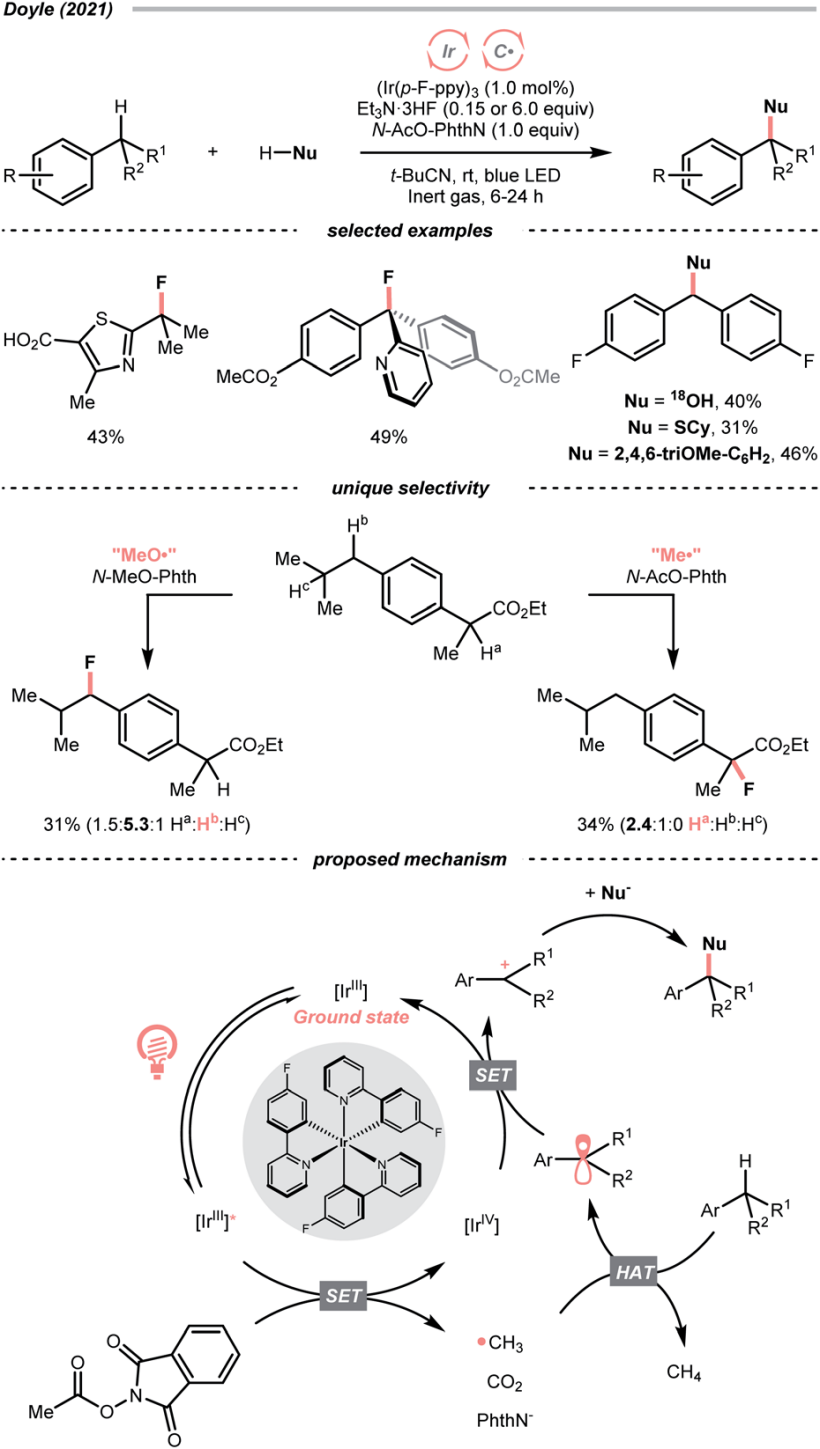
Methyl radical-mediated C(sp^3^)–H functionalisation.

Very recently, Doyle's group documented a methyl radical (Me˙)-mediated C(sp^3^)–H fluorination using *N*-acetyloxyphthalimide as Me˙ precursor and triethylamine trihydrofluoride (Et_3_N·3HF) as the fluoride (F^−^) source under radical-polar crossover mechanism (Scheme 20).^74^ In their tentative catalytic cycle, *N*-acetyloxyphthalimide was reduced to Me˙ by excited Ir(iii), with the concurrent release of CO_2_ and phthalimide. The alkane substrate underwent HAT with the Me˙. Afterwards, the generated R˙ was transformed into a carbocation, which was intercepted by the F^−^ to give the fluorinated product. Distinct HAT selectivity toward electron-deficient C(sp^3^)–H bonds was observed with the nucleophilic Me˙, which was complementary to the electrophilic radicals. Besides fluorination, C(sp^3^)–H functionalisations with different nucleophiles such as water, alcohol, chloride, azide, thiol, and electron-rich arene were also demonstrated under slightly modified conditions.

### 2.6. Miscellaneous examples

C–H bond oxidation followed by deprotonation is another R˙ generation pathway that resembles the HAT process. Unlike conventional HAT, wherein HAT agent was required, this kind of formal HAT process mandated strong oxidants or unique mechanisms to realise the stepwise R˙ generation.

**Scheme 21:**
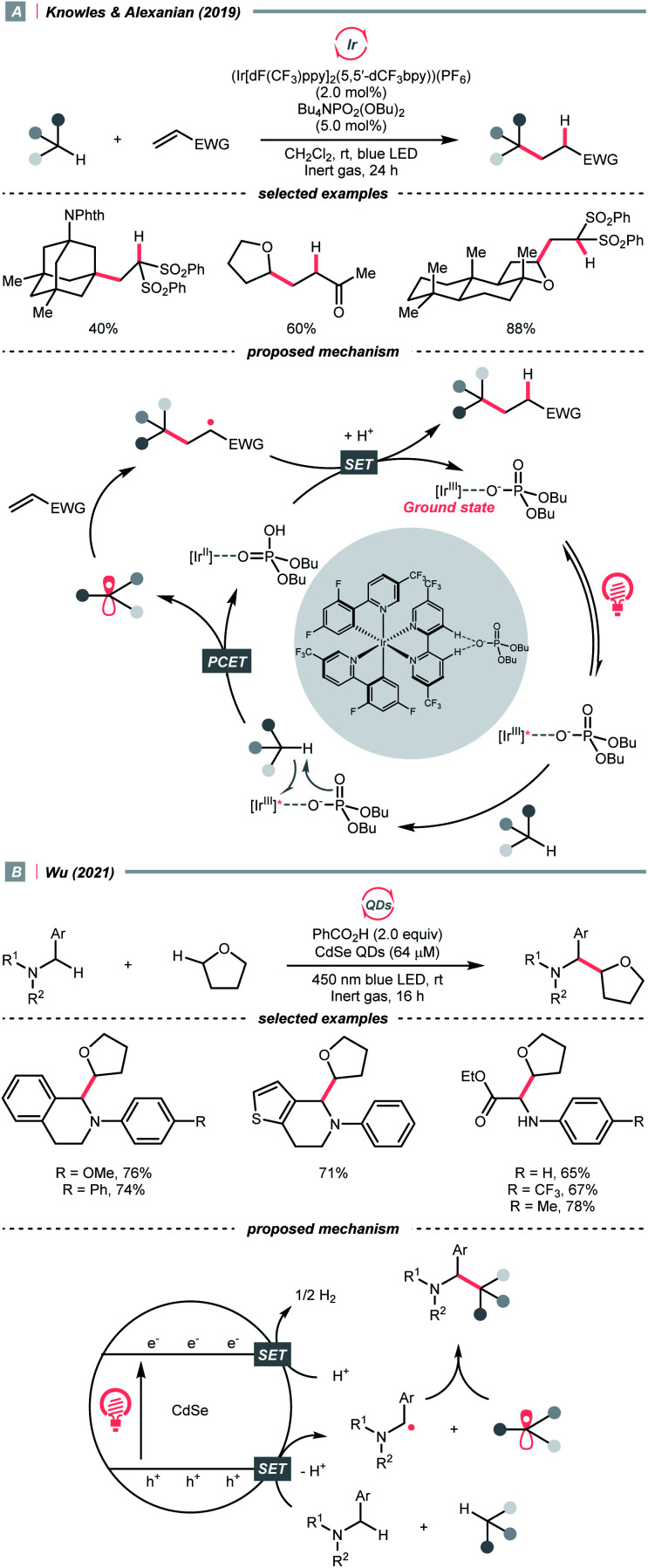
Forming R˙ through C(sp^3^)–H oxidations.

In 2019, the team of Knowles and Alexanian disclosed an unprecedented, intermolecular multisite-PCET (MS-PCET) interaction of the C(sp^3^)–H bond in the unactivated alkane with a noncovalent complex assembled from the Ir(iii) polypyridyl catalyst and an organic phosphate base (PO_2_(OBu)_2_^−^) (Scheme 21A).^75^

Detailed spectroscopic studies supported the ion-pairing between the Ir*(iii) (*E*^red^_1/2_ Ir*(iii)/Ir(ii) = +1.72 V *vs.* SCE in MeCN), and PO_2_(OBu)_2_^−^ (*E*^red^_1/2_ ≥ +2.02 V *vs.* SCE in MeCN) and precluded the possibility of the direct electron-transfer mechanism. Such an association decreased the molecularity of the elementary C–H cleavage step, which facilitated the concerted transfers of proton and electron. Various C(sp^3^)–H bonds were transformed into R˙ for the Giese reaction, with observed selectivity consistent with their bond dissociation free energies (BDFEs).

When exploiting the heterogeneous cadmium selenide quantum dots (CdSe QDs), Wu's group disclosed another new mechanism that directly activates C(sp^3^)–H bonds under blue light irradiation (Scheme 21B).^76^ Due to the large surface area to accommodate reactive species, two types of electron-rich α-heteroatomic C(sp^3^)–H bonds, in ether and 2-aryl tetrahydroisoquinoline (THIQ), respectively, were simultaneously oxidised by the holes (h^+^s) on QDs, forming two different R˙ and setting the stage for subsequent radical–radical cross-couplings. Synergistically, the electrons distributed on CdSe were consumed *via* H_2_ evolution, accomplishing the CDC reaction. This powerful QD material was also used in other radical transformations by the same group, which showed fruitful reactivities in Minisci alkylation^77^ and radical thiolation.^78^

**Scheme 22:**
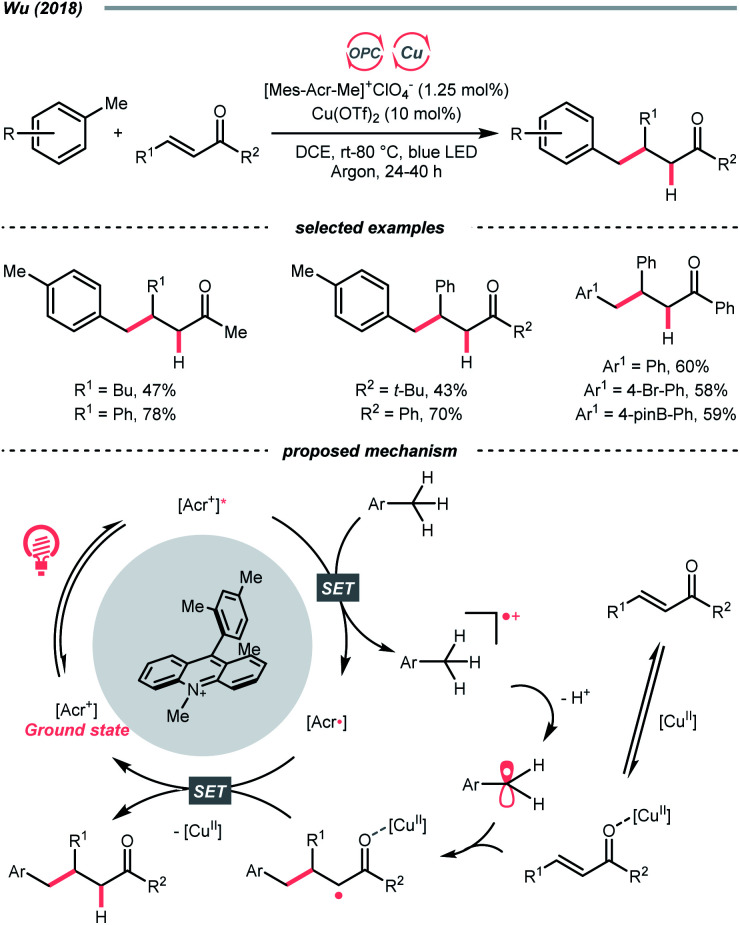
Photocatalytic benzylic C(sp^3^)–H oxidation.

The R˙ production *via* the SET/deprotonation pathway of benzylic C–H bonds in toluene derivatives could be facilitated by the initial oxidation of their arene moieties. Along this line, Chen and Wu *et al.* documented a Giese reaction between toluene and chalcone derivatives with an acridinium photocatalyst and a Cu(ii) Lewis acid catalyst (Scheme 22).^79^ In the tentative mechanism, the excited acridinium (
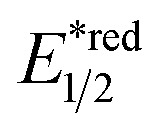
 = +2.06 V *vs.* SCE in MeCN) oxidised the toluene (*E*^red^_1/2_ = +2.36 V *vs.* SCE in MeCN) to a radical cation, in which the spin centre might initially reside in the arene, then shifted to the benzylic position after deprotonation. Despite the uphill SET process, which was crucial for breaking the benzylic C–H bond since its acidity was pronouncedly enhanced after arene oxidation, some innovative techniques (*e.g.*, “stop-flow” micro-tubing (SFMT) reactors) could expedite the electron transfer.

## 3. C–C cleavage

C–C bonds are common skeletons in organic molecules, which partially explains the high diversity of types of R˙ precursors that involve C–C cleavage to form R˙. Since C–C bonds are relatively inert, driving forces such as small molecule extrusion (*i.e.*, CO_2_, CO, acetone), strain release, and aromatisation are frequently considered to facilitate the C–C bond cleavage. In this regard, oxygen-containing molecules, such as carboxylic acids, aldehydes and alcohols, and some dearomatised compounds, are versatile R˙ precursors that undergo photoscission of C–C bonds.

### 3.1. C–C cleavage of carboxylic acid derivatives

Alkyl carboxylic acids are naturally abundant and bench-stable.^80^ Although CO_2_ extrusion offers the enthalpic advantage to elicit R˙ from carboxylic acids, high temperatures and strong oxidants, and sometimes the presence of transition metals, were required in traditional decarboxylation methods. Fortunately, advancements in photocatalysis have allowed decarboxylative R˙ generation to procced under mild conditions.^81^

**Scheme 23:**
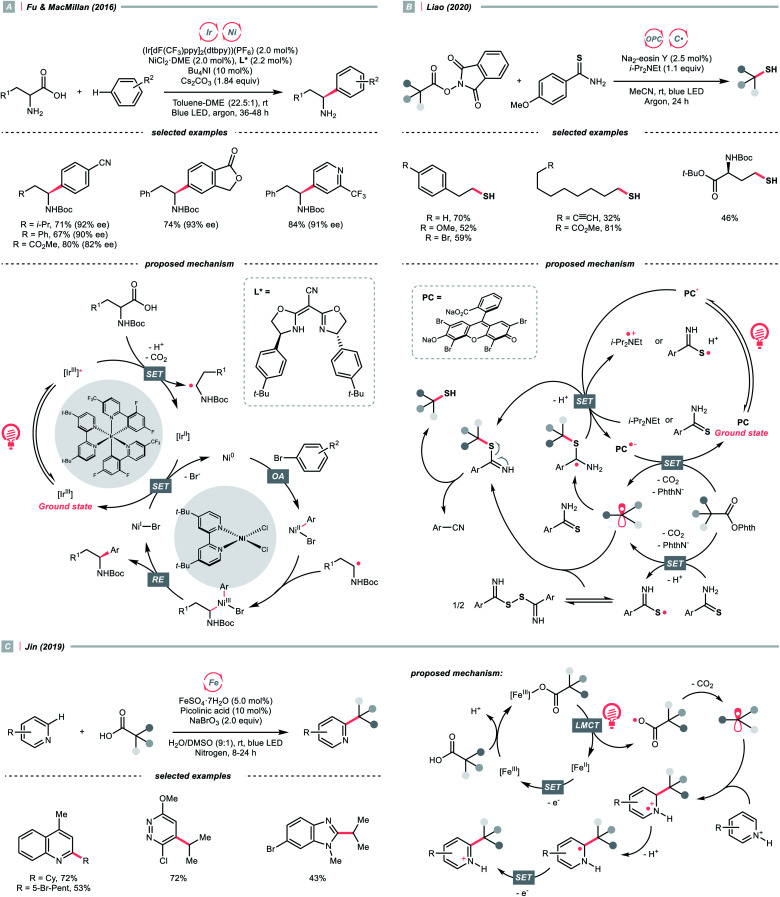
R˙ formation *via* carboxyl radical intermediates.

In 2014, Doyle, MacMillan and their co-workers reported the seminal work of metallaphotoredox catalysis by combining photoredox catalysis with nickel catalysis for decarboxylative arylation of α-amino acids with aryl halides,^82^ which revolutionised the conventional design for transition metal-catalysed cross-couplings.^5,83^ Later in 2016, the team of Fu and MacMillan upgraded this dual catalysis protocol to an asymmetric version using a chiral bisoxazoline ligand (Scheme 23A).^84^ In this metallaphotoredox mechanism, single-electron oxidation of the carboxylate (for *tert*-butyl carbamoyl Boc-Pro-OCs, *E*^red^_1/2_ = +0.95 V *vs.* SCE in MeCN) with the excited Ir(iii) (*E*^red^_1/2_ Ir*(iii)/Ir(ii) = +1.21 V *vs.* SCE in MeCN) generated a carboxyl radical, which decomposed into an R˙ *via* releasing CO_2_. Meanwhile, the chiral aryl nickel(ii) bromide generated from the oxidative addition of nickel(0) to aryl bromide trapped the R˙, followed by the reductive elimination to afford the α-arylated amines in moderate to good yields with good to excellent enantioselectivities.

By strategically designing some carboxylic acid derivatives, the decarboxylation could also be put into a reduction-initiated manifold. For example, alkyl diacyl peroxides ((RCOO)_2_) that were synthesised from aliphatic acids could reverse the redox properties of their parent acids and favour the reductive decarboxylation.^85^ However, they are often chemically labile and potentially explosive, which cause safety concerns. Pioneered by Okada, and later revisited by Baran, Glorius and others, the carboxylic acid was converted into the relatively stable *N*-(acyloxy)phthalimides, which were repurposed radical alkylating reagents and so-called redox-active esters (RAEs).^86–92^ Since these pioneering research, a diverse range of radical alkylation reactions have been published. One recent photocatalytic example was Liao's report of decarboxylative thiolation of *N*-(acyloxy)phthalimides utilizing Na_2_-eosin Y in the presence of sulfur donor and amine reductant (Scheme 23B).^93^ Mindful of the sensitivity of thiol products toward oxidative conditions, reductive generation of R˙ from photocatalytic decarboxylation could effectively avoid the undesired sulfur oxidation. Under the photoreduction conditions, RAE underwent a SET/fragmentation sequence to release CO_2_ and phthalimide, giving the R˙ to be thiolated subsequently. To be noticed, other than these carboxylic acid derivatives, using anhydride^94^ and pyridine *N*-oxide^95^ were also reported for C(sp^3^) radical generation with photoredox catalysis.

Apart from undergoing the SET pathway, aliphatic acid could be oxidised to form R˙ *via* LMCT of the photoexcitable transition metal carboxylate. In 2019, Inspired by the photoinduced iron(iii)-mediated decarboxylative Minisci alkylation by Sugimori,^96^ Jin's group advanced an iron-catalysed version with the picolinic acid ligand under visible light (Scheme 23C).^97^ In their proposed reaction mechanism, iron(ii) carboxylate was oxidised by BrO_3_^−^ to iron(iii), which, upon photoexcitation, was susceptible to Fe–O bond homolysis. Decarboxylation ensued, producing the R˙ for the sequential Minisci alkylation.

The picolinic acid ligand was crucial in altering the photophysical properties of the iron catalyst since the desired reactivity was inhibited in its absence. To be noticed, with the same iron/ligand set, Jin's group also applied this decarboxylative R˙ generation strategy for conjugate addition to construct C–C and C–N bonds under redox-neutral conditions.^98^

### 3.2. C–C cleavage of carbonyl derivatives

The decarbonylation of carbonyl compounds is another common way to generate an R˙, wherein the R˙ precursor, acyl radical, is frequently obtained from aldehydes through HAT.^99,100^

**Scheme 24:**
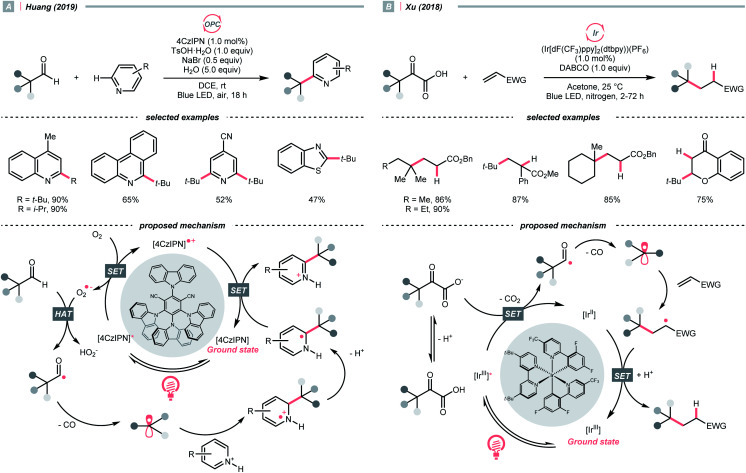
R˙ formation from acyl radical intermediates.

In 2019, Huang and his co-workers developed a photocatalysed decarbonylative Minisci alkylation with aldehydes under air (Scheme 24A).^101^ In their critical R˙-generating steps, the O_2_ in the atmosphere was reduced by photoexcited 4CzIPN, generating a superoxide radical anion (O_2_˙^−^). This oxy radical could perform HAT at the formyl C–H to give an acyl radical, followed by a radical decarbonylation to release CO and give an R˙.

Alternatively, acyl radicals could derive from an oxidative decarboxylation reaction of α-ketoacids, followed by the decarbonylation to give the R˙. Capitalizing on this special class of oxocarboxylic acids, in 2018, Xu and co-workers described a Giese reaction with α-ketoacids under photoredox conditions (Scheme 24B).^102^ In the presence of a base, the α-oxocarboxylate (for pyruvic acid, *E*^red^_1/2_ = +1.03 V *vs.* SCE in DMSO) was oxidised by the excited (Ir[dF(CF_3_)ppy]_2_(dtbpy))(PF_6_) (*E*^red^_1/2_ Ir*(iii)/Ir(ii) = +1.21 V *vs.* SCE in MeCN) into carboxyl radical, delivering the R˙ with concurrent evolution of CO_2_ and CO. However, the acylation product dominated when targeting the less stabilised 2° and 1° radical, or the bulky adamantly one. Fortunately, alkylation with these R˙ precursors could be more efficient toward alkylation with refluxing.

### 3.3. C–C cleavage of alcohol derivatives

SET of aliphatic alcohols could also effect the R˙ generation *via* different C–C bond-breaking pathways. Among them, dehydroxymethylation of β-substituted alcohols could give the R˙ after decarbonylation.

**Scheme 25:**
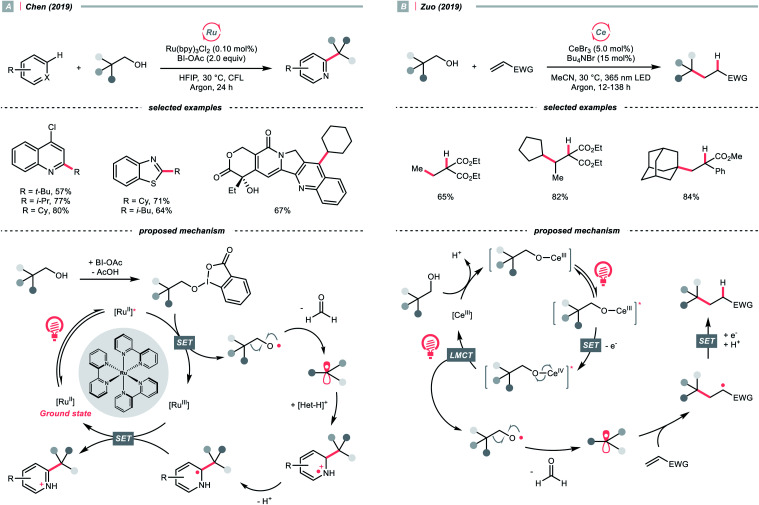
R˙ generation from alcohol and its derivatives.

To sidestep the sluggish oxidation of the –OH group, Chen's group converted the alcohol into alkoxybenziodoxolone (BI–OCH_2_R) through condensation with 1-acetoxy-1,2-benziodoxol-3-(1*H*)-one (BI-OAc). The *in situ* assembled BI-OCH_2_R underwent reductive SET by the excited Ru(ii)-photocatalyst and produced an RCH_2_O˙, which delivered an R˙ for Minisci alkylation after β-scission and removal of formaldehyde (Scheme 25A).^103^

Alternatively, Zuo and his co-workers demonstrated a Ce-catalysed dehydroxymethylative functionalisation reaction with β-substituted alcohols, wherein the key alkoxy radicals were generated by the LMCT of photoexcited Ce(iv) alkoxide (Scheme 25B).^104,105^ Similar to Chen's case, β-cleavage of the C–C bonds in alkoxy radical give the R˙, which could engage in alkylation, alkenylation, oxygenation, and hydrogenation cross-couplings with alkyl halides. The catalyst turnover mechanism varied with the light sources as cerium could catalyse the dehydroxymethylative Giese reaction alone under 365 nm. However, it dictated the presence of 9,10-diphenylanthracene as co-catalyst to accomplish the same reaction with 400 nm irradiation.

### 3.4. C–C cleavage *via* strain release

Strain release of congested cyclic compounds could be induced by ring-opening radical translocation or radical addition, which were two common pathways to generate the R˙ from strained structures.

NCR resulting from SET could enable R˙ generation *via* breaking neighbouring C–C bonds. Especially, ring-opening of cyclic iminyl radical intermediates, which are commonly derived from oxime derivatives, are useful in synthesizing alkyl nitriles.

**Scheme 26:**
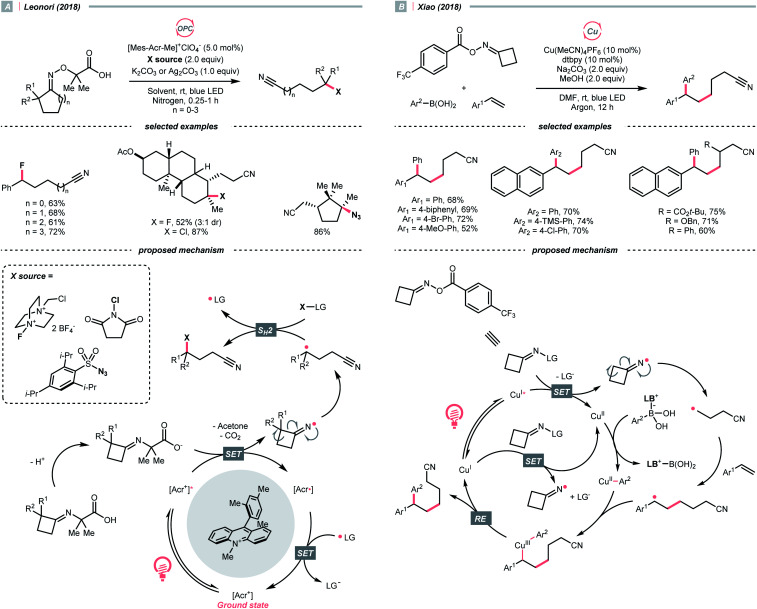
R˙ formation *via* iminyl radical-induced ring-openings.

In 2018, Leonori's group showcased a photoredox synthesis of remotely functionalised alkyl nitriles using *O*-alkylated oximes derived from carboxylic acid (Scheme 26A).^106^ With the equimolar carbonate base, the carboxylate was oxidised by Fukuzumi's catalyst. Following CO_2_ and acetone extrusion, the cyclic iminyl radical quickly fragmented into the R˙ with a terminal nitrile group for the subsequent fluorination, chlorination, or azidation. Ring-opening of four-membered rings was facile, while the unstrained five to seven-membered ones required two α-methyl groups or one α-phenyl substituent to facilitate C–C cleavage. By intercepting the cyanated R˙ with nickel catalysis, radical ring-opening arylation, vinylation and alkylation cascades were achieved by the same group with corresponding carbon electrophiles.^107^

Reductive SET of engineered oximes could also furnish the iminyl radicals for R˙ generation *via* ring-opening. In the same year, Xiao *et al.* exemplified a visible-light-induced copper-catalysed styrene difunctionalisation reaction with oxime esters and aryl boronic acids (Scheme 26B).^108^ In their proposed mechanism, the excited copper(i) reduced the *O*-benzoyl oxime ester and triggered its N–O cleavage and β-scission to give the cyanated R˙. Meanwhile, copper(ii) exchanged its ligand with boronic acid to form an aryl Cu(ii) species. The R˙ was trapped by the styrene and subsequently by the aryl copper(ii) intermediate, which gave the 1,2-difunctionalised alkane after a facile Cu(iii) reductive elimination and closed the copper catalytic cycle.

**Scheme 27:**
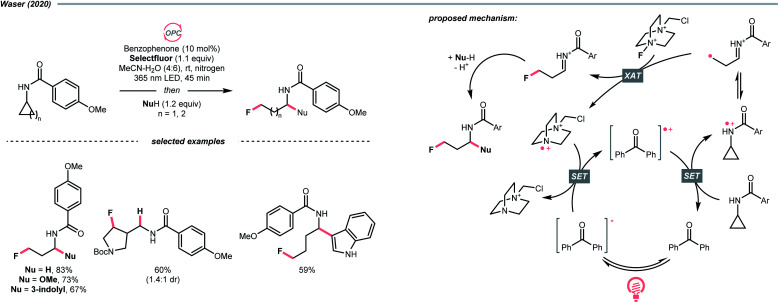
Generating R˙ from oxidative ring-opening of amide.

Aligned with iminyl radical, amidyl radical is another NCR that could generate R˙ by breaking the C–C bond in cyclic structures. In 2020, Waser's laboratory developed a ring-opening strategy to achieve oxidative difunctionalisations of *N*-cyclopropyl and *N*-cyclobutyl amides with Selectfluor as the fluorine source and benzophenone as the photocatalyst (Scheme 27).^109^ Mechanistically speaking, a strongly oxidising benzophenone radical cation (*E*^red^_1/2_ (BP^+^/BP) = +2.37 V *vs.* SCE in MeCN) was formed by photooxidation with Selectfluor or its amino radical cation (for benzophenone, *E*^red^_1/2_ (BP^+^/BP*) = −0.62 V *vs.* SCE in MeCN; for Selectfluor, *E*^red^_1/2_ = +0.33 V *vs.* SCE in MeCN and for Selectfluor-derived *N*-radical cation, *E*^red^_1/2_ = +0.79 V *vs.* SCE in MeCN). With this potent oxidising species, the amide (for cyclopropyl amide, *E*^red^_1/2_ = +1.67 V *vs.* SCE in MeCN) was turned into an amido radical cation, then an iminium-containing R˙ after ring-opening. Selectfluor serves as both fluorine source and oxidant for benzophenone regeneration. Stepwise addition of a nucleophile, including alcohol, hydroperoxide, thiol, trifluoroborate, and electron-rich arene to the fluorinated imine intermediates, could accomplish the corresponding difunctionalisations.

Besides C–C cleavage of cyclic oxime and amides [1.1.1]propellane, a simple and strained bicyclic molecule, could directly interact with a radical species to cleave its central C–C bond and afford a C(sp^3^) radical. Since [1.1.1]propellane has been demonstrated as a useful synthon to access disubstituted bicyclo[1.1.1]pentane (BCP),^110–115^ a high-value three-dimensional bioisostere for phenyl, alkynyl, and *tert*-butyl groups in drug designs,^116,117^ its synthetic application has received attention in photocatalysis.

**Scheme 28:**
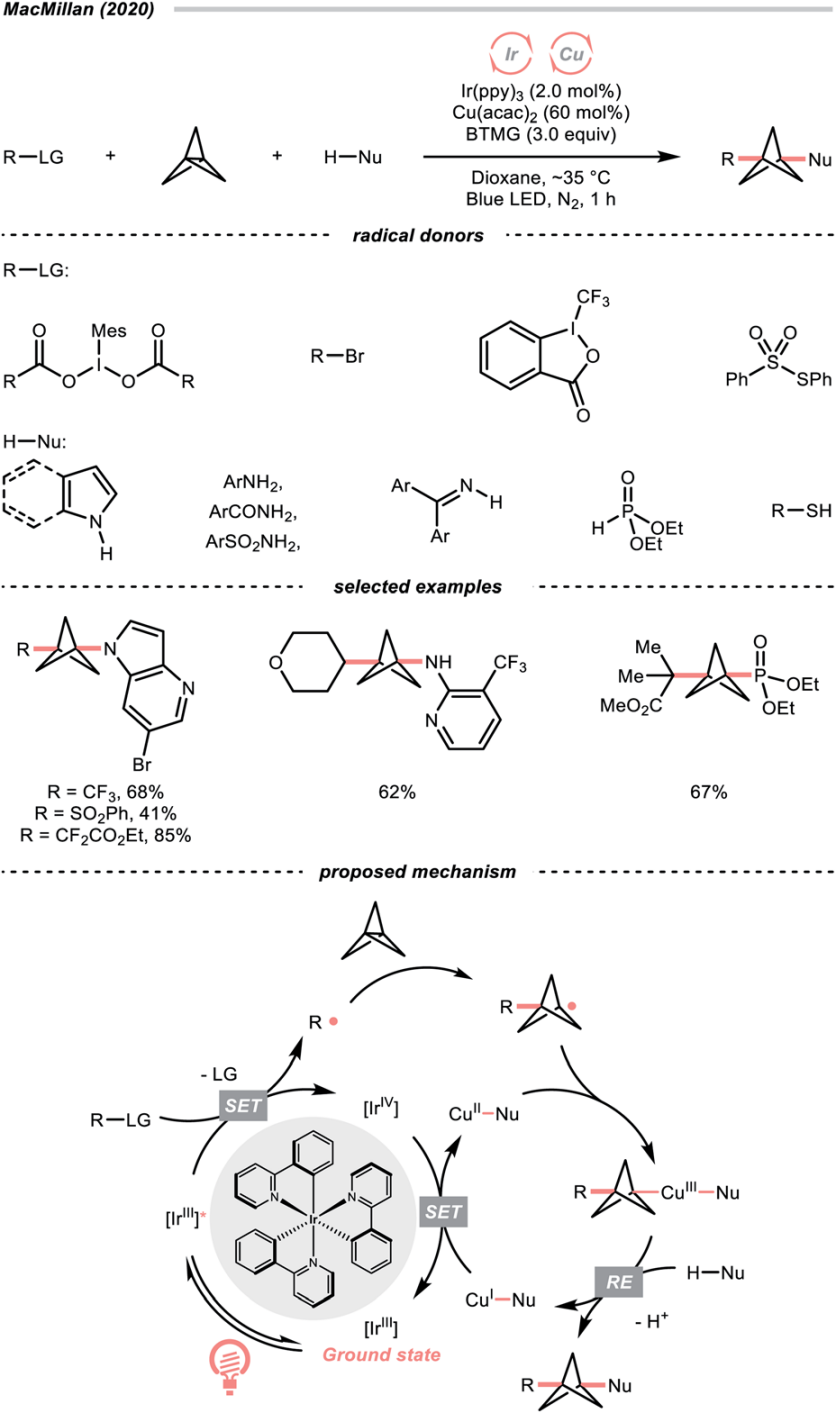
Radical addition to [1.1.1]propellane.

Driven by its great utility, MacMillan's group reported a difunctionalisation reaction of [1.1.1]propellane by merging copper catalysis and iridium photocatalysis (Scheme 28).^114^ In their tentative mechanism, the excited Ir(iii)-photocatalyst (*E*^red^_1/2_ Ir(iv)/Ir*(iii) = −1.73 V *vs.* SCE in MeCN) reduced the iodonium dicarboxylate (for the 4-tetrahydropyranyl one, *E*^red^_1/2_ = −0.82 V *vs.* SCE in MeCN), alkyl bromide, or sulfonothioate *via* SET to generate a C- or S-centred radicals, respectively, which could readily combine with [1.1.1]propellane to give a bicyclo[1.1.1]pentyl radical (R˙) through strain release. By leveraging copper catalysis, the R˙ was then coupled with various nucleophiles such as electron-rich arenes, anilines, and sulfonamides, offering 1,3-disubstituted BCPs through the facile R–Cu(iii)–Nu reductive elimination.

### 3.5. C–C cleavage *via* aromatisation

Dearomatised arenes and heteroarenes are reducing and tend to rearomatise. With such a strong driving force, various alkylating reagents in this class are designed to release the R˙ *via* rearomative C–C cleavage under photocatalytic conditions.

**Scheme 29:**
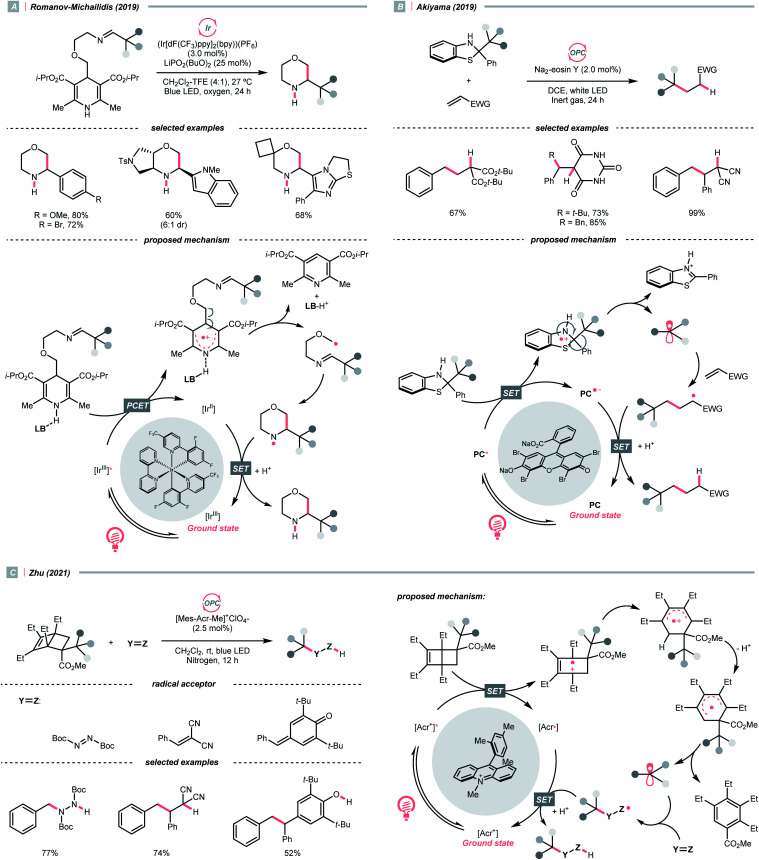
Aromatisation-induced R˙ extrusions.

Aza-cyclohexadienyl radical is a special amino radical that mostly stems from the oxidative SET with dihydropyridines (DHPs). Bearing a strong tendency toward aromatisation *via* C–C cleavage at its *para*-position, 4-alkylated DHPs are viable R˙ sources. Taking a C4-iminoalkylated Hantzsch ester (*E*^red^_1/2_ = +1.01–1.23 V *vs.* SCE in MeCN) as the starting material, Romanov-Michailid *et al.* elaborated the synthesis of several 3-aryl morpholines *via* R˙ cyclisation (Scheme 29A).^118^ Mechanistically, PCET between the (Ir[dF(CF_3_)ppy]_2_(bpy))(PF_6_) (*E*^red^_1/2_ Ir*(iii)/Ir(ii) = +1.32 V *vs.* SCE in MeCN) and a Lewis base was believed to be a key step in this O_2_-mediated cyclisation protocol. Ring-closure of the R˙ with its imine tail and the subsequent reductive quenching will give the morpholine as the desired product. Interestingly, pioneered by Melchiorre's group, several photocatalyst-free alkylation protocols with direct excitation of Hantzsch esters were also documented.^119,120^

Aside from DHPs, other heterocycles and carbocycles that could enforce the rearomative C–C cleavage to give the R˙ were reported. For instance, Akiyama's group used C2-alkylated 2-phenyl dihydrobenzothiazoles (
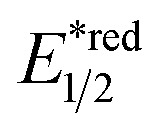
 = +0.70 V *vs.* SCE in MeCN) for photoinduced radical alkylation with electron-deficient alkenes as the acceptors and Na_2_-eosin Y as the photocatalyst (Scheme 29B, *E*^red^_1/2_ (eosin Y*/eosin Y˙^−^) = +0.83 V *vs.* SCE in 1 : 1 MeCN–H_2_O).^121^ Very recently, Zhu's group reported a novel alkylated bicyclo[2.2.0]hexene reagent (for benzylation, *E*^red^_1/2_ = +1.59 V *vs.* SCE in MeCN) for 1,2-addition to azodicarboxylate and electron-poor alkene, as well as 1,6-addition to *para*-quinone methides with [Mes-Acr-Me]^+^ClO_4_^−^ (Scheme 29C, E*_1/2_^red^ = +2.06 V *vs.* SCE in MeCN).^122^ In both cases, single-electron oxidation of R˙ precursors afford radical cations, which split into a (hetero)arene and an R˙. The latter was submitted to typical photocatalysed Giese-type pathways to give the alkylated products.

### 3.6. C=C π-bond cleavage

Radical addition to the alkenyl π-bond is a method to generate a C(sp^3^) radical that could increase the structure and functional complexities of incoming radical species. In some cases, such a radical translocation process could expedite the new radical generation since the radical adducts are usually more stable. Furthermore, radical philicity reversal is also a common purpose by introducing these primary radical acceptors, which could be highly useful in organic synthesis and polymer chemistry.^123,124^

The addition of R˙ to electronically matched double bonds is commonly seen in the literature of alkene hydrofunctionalisations and difunctionalisations, some of which have been reviewed in previous examples. Instead, adding heteroatomic radicals to the olefins and merging the new R˙ in different radical cascade reactions was rarely seen and will be exemplified below.

**Scheme 30:**
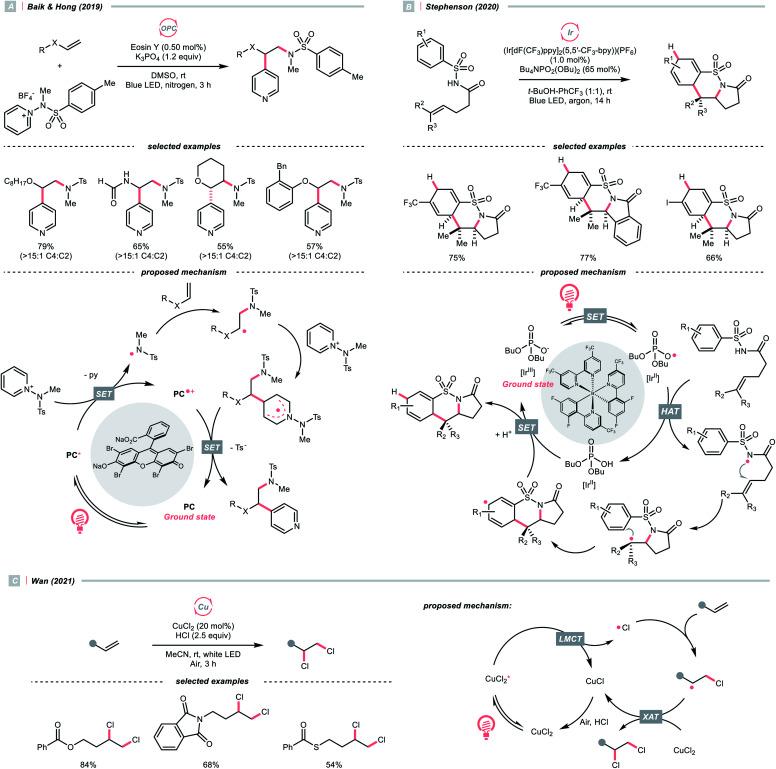
Photocatalysed R˙ formation *via* radical addition to C=C bonds.

As an intermolecular example, in 2019, Baik and Hong's team made a breakthrough on C4-selective heteroarene alkylation, which is a long-lasting challenge when both the heteroaromatic C2 and C4 sites are non-substituted (Scheme 30A).^125^ The key for such a unique selectivity was the usage of *N*-(methyltosyl)aminopyridinium salts (*E*^red^_1/2_ = −0.70 V *vs.* SCE in MeCN), which provided the NCR upon single-electron reduction by the excited eosin Y (*E*^red^_1/2_ (eosin Y*/eosin Y˙^+^) = −1.11 V *vs.* SCE in 1 : 1 MeCN–H_2_O) and steered the R˙ formed from the NCR and alkene toward its C4 position *via* steric and some secondary interaction. The observed high regioselectivity complied with the density functional theory (DFT) calculation, suggesting an N–N bond cleavage mechanism of the *N*-aminopyridinium to give the final product and a new NCR. It was worth mentioning that after the combination of the electrophilic NCR and the electron-rich alkene, a nucleophilic R˙ was generated for the alkylation of electron-poor pyridinium, which demonstrated the significance of matching the polarity and the versatility of alkene in inversing the radical polarity.

A similar reaction sequence in an intramolecular fashion could lead to different reaction outcomes. Stephenson's laboratory reported a photoinduced dearomative cyclisation of alkenyl *N*-arylsulfonamides with (Ir[dF(CF_3_)ppy]_2_(5,5′-CF_3_-bpy))(PF_6_) and a HAT agent Bu_4_NPO_2_(OBu)_2_ (Scheme 30B).^126^ At first, a ground-state Ir(iii) and dibutyl phosphate aggregation, as proposed by Alexanian and Knowles (Scheme 21A), was suspected of enabling the NCR generation. Nonetheless, careful spectroscopic studies suggested the MS-PCET and deprotonation/oxidation mechanism to generate the NCR were less likely in this case. Instead, after light-induced SET between the Ir(iii) and phosphate, an oxy radical departed and performed the HAT with the sulfonamide N–H. NCR addition to the terminal alkene formed an R˙, which then performed an intramolecular radical cyclisation to the pendent arene. Subsequent SET/protonation steps of the cyclohexadienyl radical delivered the dearomatised product.

In addition to NCRs, the addition of chlorine radical to aliphatic alkene could also effect the alkyl radical generation, as demonstrated by Wan's group (Scheme 30C).^127^ In this case, a vicinal dichlorination was succeeded, wherein CuCl_2_ catalyst and HCl supplemented the chlorine sources under ambient conditions. However, styrene 1,2-dichlorination was compromised by some side reactions, which could be suppressed using over-stoichiometric CuCl_2_.

**Scheme 31:**
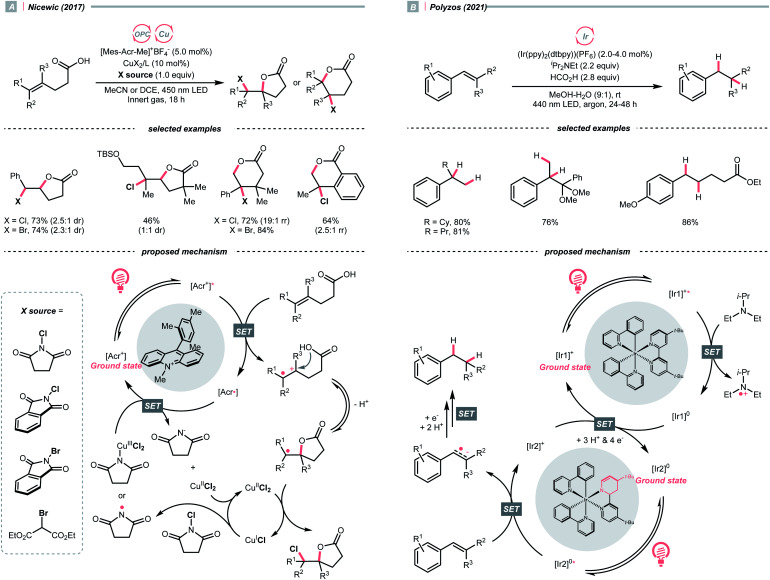
Photocatalysed SET-induced C=C cleavages.

In contrast to the radical addition mechanism above, some novel photocatalysis that could provide highly oxidising or reducing environments has been used to generate R˙ *via* direct SET on alkenes, which was trapped by some nucleophiles or electrophiles, respectively. Single-electron oxidation of a C=C bond gives a C–C radical cation, simultaneously serving as an R˙ source and an electrophile for alkene difunctionalisations. Under this reaction paradigm, Nicewicz and his co-workers disclosed their seminal discovery on photocatalysed intramolecular hydroetherification of alkenols in 2012.^128^ Later, several intra- and intermolecular alkene hydrofunctionalisations followed.^129–131^ In 2017, they advanced this protocol by merging Cu(ii) catalyst and electrophilic halogen sources for halolactonisation of unsaturated fatty carboxylic acids (Scheme 31A).^132^ The strongly oxidising [Mes-Acr^+^-Me]ClO_4_^−^ (
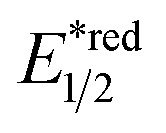
 = +2.06 V *vs.* SCE in MeCN) played a pivotal role in this alkene radical cation chemistry since the reduction potential of alkenes were often beyond the reach of many common photocatalysts.

On the other hand, single-electron reduction of the alkene will leave a radical anion. In 2021, Polyzos published a photocatalysed styrene reductive hydrofunctionalisation with proton or ketone as electrophiles and amine as the sacrificial reductant (Scheme 31B).^133^ Interestingly, a multiphoton tandem photoredox cycle of the Ir-photocatalyst [Ir(ppy)_2_(dtbbpy)]PF_6_ was proposed. Central to this method was the photocatalytic generation of a new Ir species with a partially reduced bipyridine ligand, which could initiate another photocatalytic cycle and deliver higher-energy electrons to overcome the high reduction barrier of styrene (typically, *E*^red^_1/2_ = <−2.0 V *vs.* SCE in MeCN).

## 4. C–X cleavage

Exploring versatile aliphatic derivatives as R˙ sources can offer more synthetic opportunities for retrosynthetic analysis. Since each type of C–X bond has distinct chemical and photophysical properties, they are often paired with unique activation strategies, which are useful in different synthetic settings. With considerable efforts devoted to the field of photocatalysis, various alkylating reagents, including aliphatic halides, alcohols, amines, boronic acids, and others, have been demonstrated as effective R˙ sources.

### 4.1. C–N cleavage

While photocatalytic radical C–N bond formation is of tremendous interest in both academic and industrial settings, C–N bond cleavage typifies an equally important direction, which played an indispensable role in R˙ generation strategies within the SET manifold.

**Scheme 32:**
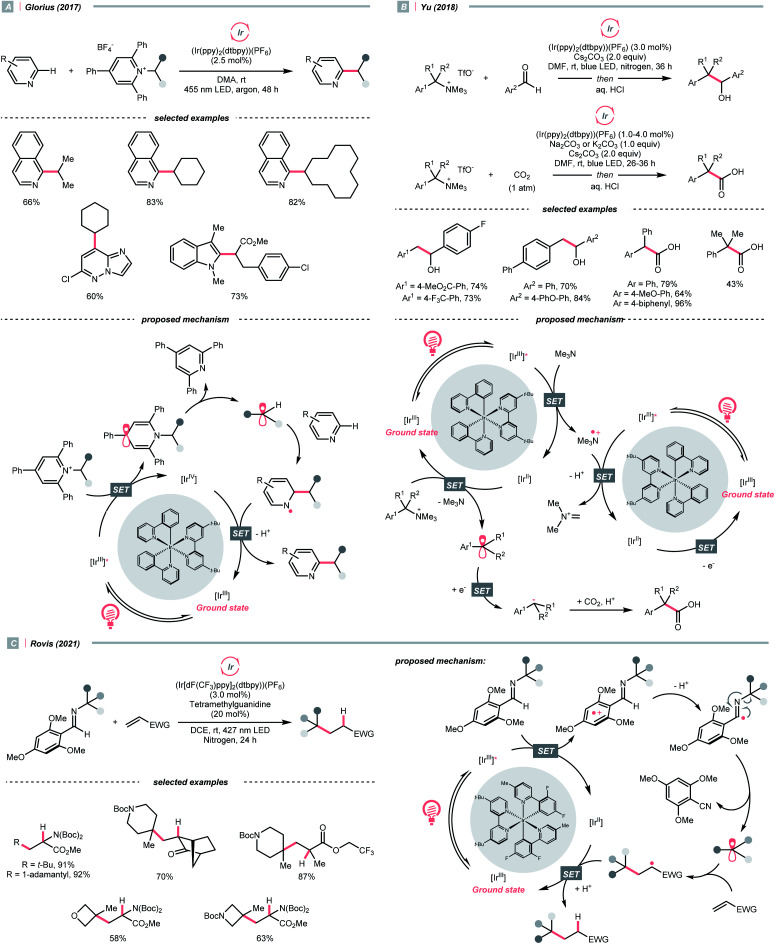
R˙ generation *via* C–N cleavage of quaternary nitrogen salt.

Quaternary ammonium or pyridinium salts, featuring highly polarised C–N bonds because of the cationic nitrogen, are widely used in radical alkylations *via* single-electron reduction. In 2017, Glorius *et al.* described a novel Minisci alkylation reaction with Katritzsky salts (R-TPP) under iridium photocatalysis (Scheme 32A).^134^ Mechanistically, the electron-deficient pyridinium (for Et-TPP, *E*_1/2_ = −0.93 V *vs.* SCE in DMF) accepted an electron from the excited Ir(iii) (*E*^red^_1/2_ Ir(iv)/Ir*(iii) = −0.96 V *vs.* SCE in MeCN) and formed a dihydropyridine radical, whose C–N fragmentation will engender the R˙ for addition to the neutral heteroarene. Notably, Brønsted acids, typical additives in Minisci alkylation conditions, were absent in Glorius's case. Moreover, electron-rich heteroarenes were accommodated in this photocatalytic approach, in which various proteinogenic amino acids were masked as R˙ sources *via* radical deamination.

For the Katritzsky salt-based method above, the pyridinium-forming process is limited to the condensation with less hindered primary amines.^135–138^ In contrast, quaternary ammonium salts could originate from nearly all types of amines *via* exhaustive alkylation, typically with methyl iodide or triflate. Using these bench-stable R˙ sources, Yu's group achieved the photocatalysed reductive cross-electrophile coupling reactions with alkyl ammonium salts and benzaldehydes or CO_2_ (Scheme 32B).^139^ Resembling the Katritzsky salts, a single-electron reduction of the ammonium salt (for BnNMe_3_OTf, *E*^red^_1/2_ = −1.58 V *vs.* SCE in DMF) with the *in situ* generated Ir(ii) (*E*^red^_1/2_ Ir(iii)/Ir(ii) = −1.49 V *vs.* SCE in DMF) could break the C–N bond and produce the R˙. The NMe_3_ released from ammonium decomposition served as a reductant to reduce Ir(iii) and regenerate the Ir(ii), which further reduced R˙ into a carbanion to react with benzaldehyde or CO_2_, giving benzyl alcohols and carboxylic acids, respectively.

Besides quaternary salts, Rovis's group recently demonstrated that *N*-alkylated redox-active imines could generate R˙ under photoredox conditions (Scheme 32C).^140^ Unlike the previous case, due to the electron richness of (2,4,6-trimethoxyphenyl)methanimine (for *N-tert*-butyl one, *E*^red^_1/2_ = +1.40 V *vs.* SCE in MeCN), it was oxidised into a radical cation by the Ir*(iii) (*E*^red^_1/2_ Ir*(iii)/Ir(ii) = +1.21 V *vs.* SCE in MeCN). The generated radical cation underwent deprotonation/SCS to form an imidoyl radical, followed by the β-scission to give the R˙ with the loss of 2,4,6-trimethoxybenzonitrile. However, the concerted or stepwise mechanism of the above PCET process could not be ascertained. With careful experimentations, the authors suggested the HAT with the iminyl C–H was less likely to contribute to the R˙ formation. Additionally, it is worth noting that owing to the facile condensation of the aldehyde with sterically encumbered amine, Rovis's method could generate tertiary R˙ easily, which was difficult with the quaternary nitrogen salts.

### 4.2. C=N cleavage

**Scheme 33:**
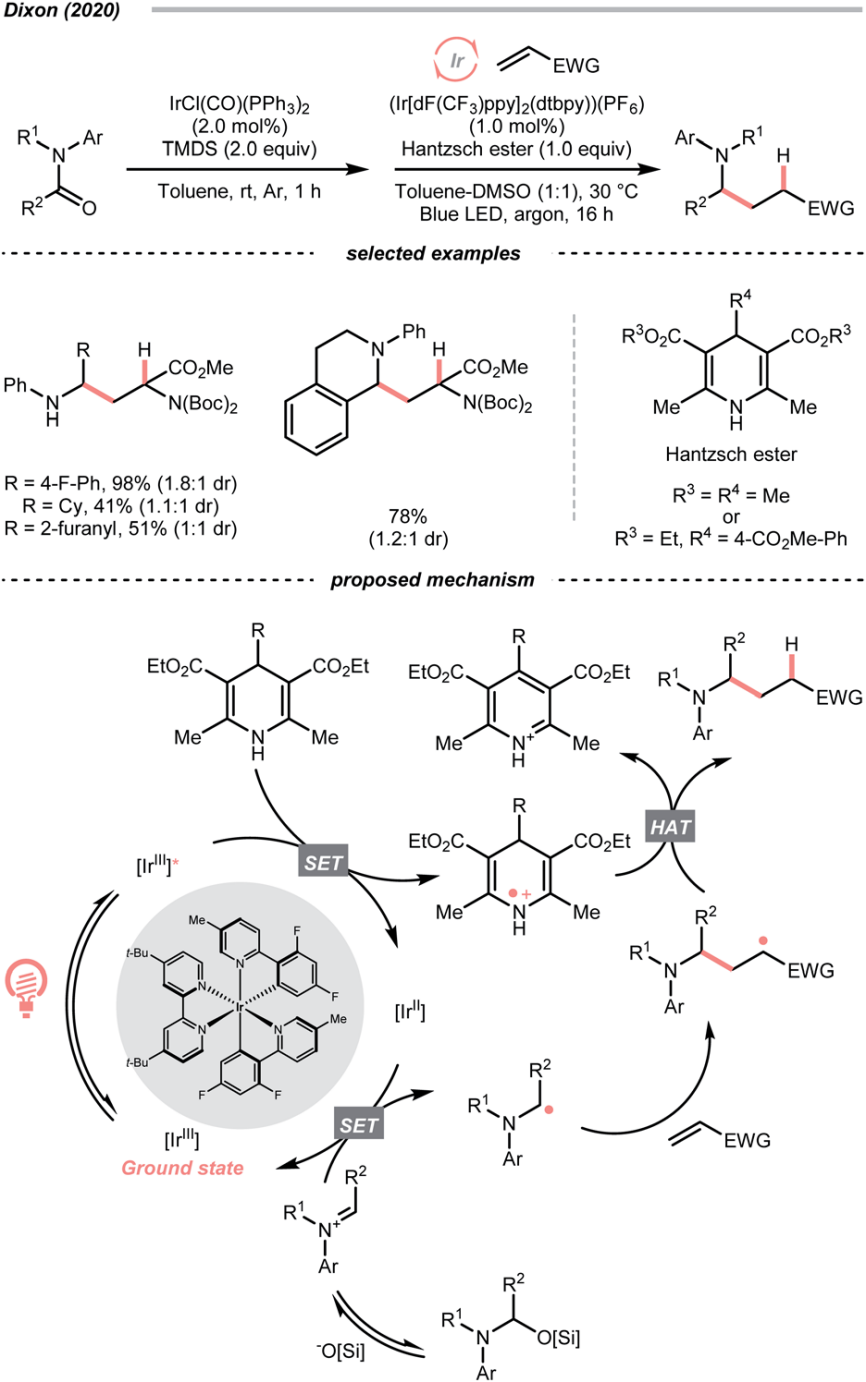
Reductive C=N π-bond cleavage of iminium salt.

Instead of breaking C–N σ-bond, cleaving the π-bond in some C=N-containing compounds could afford R˙ as well. In the case of iminiums, the group of Duarte and Dixon detailed a one-pot-two-step deoxygenative alkylation reaction of tertiary amides in 2020, with iminium ions as the key intermediates to generate α-amino radicals for Giese addition (Scheme 33).^141^ In the first step, hydrosilylation of the amide to furnish an iminium was executed with 1,1,3,3-tetramethyl disiloxane (TMDS) and Vaska's catalyst (IrCl(CO)(PPh_3_)_2_). Then, the iminium (*E*^red^_1/2_ = −0.96 V *vs.* SCE in DMSO) was subject to the reductive coupling with Ir-photocatalyst (*E*^red^_1/2_ Ir*(iii)/Ir(ii) = +1.21 V *vs.* SCE in MeCN; *E*^red^_1/2_ Ir(iii)/Ir(ii) = −1.42 V *vs.* SCE in DMSO), Hantzsch ester (for 4-phenyl ethyl ester, calculated *E*^red^_1/2_ = +0.75 V *vs.* SCE in DMSO) and Michael acceptors under photo-irradiation, which shares a similar mechanism to their developed enol ether alkylations (Scheme 40C).^142^

**Scheme 34:**
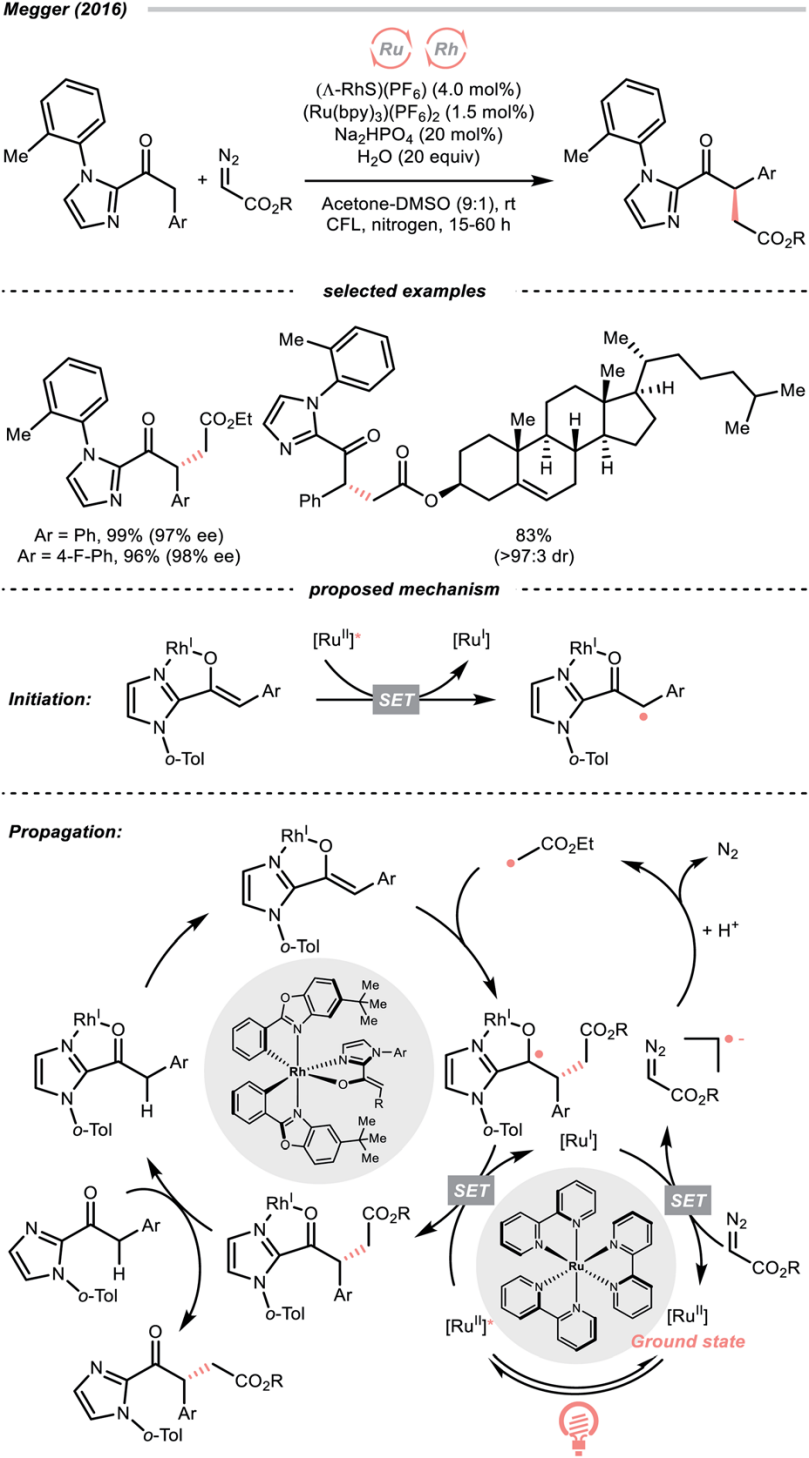
Generating R˙ from aliphatic diazo compounds.

The R˙ generation could also proceed with complete C=N cleavage of diazo compounds, which has been explored by many laboratories.^143–146^ In 2016, the group of Megger merged the Ru-photoredox catalysis with a chiral Rh(i) catalysis for asymmetric α-C(sp^3^)–H functionalisation of arylacetyl imidazoles with diazoacetate as the R˙ source (Scheme 34).^147^ In the early stage, the Rh(i) chelated the carbonyl and imidazolyl groups of the substrate and formed an enolate complex (Rh-enolate). Based on the fluorescence quenching studies, the reaction was believed to start from the SET between the Rh-enolate (with 2-acyl imidazole, *E*^red^_1/2_ = +0.50 V *vs.* SCE in MeCN) and the Ru(ii)-photocatalyst (*E*^red^_1/2_ Ru*(ii)/Ru(i) = +0.77 V *vs.* SCE in MeCN). After the initiation, albeit slightly endergonic, the single-electron reduction of the diazo substrate (for ethyl diazoacetate, *E*^red^_1/2_ = −1.55 V *vs.* SCE in MeCN) by the reducing Ru(i) (*E*^red^_1/2_ Ru(ii)/Ru(i) = −1.33 V *vs.* SCE in MeCN) will give the R˙ after protonation and N_2_ extrusion. The stereo-determining R˙ addition to the chiral Rh-enolate and the SET with Ru*(ii) closed both catalytic cycles and propagated the radical chain.

Notably, despite the proposed radical chain mechanism, a low quantum yield (*ϕ* = 0.10) was determined for the model reaction, which could attribute to some sophisticated deactivation pathways like competing light absorption and quenching effects. However, based on this dual photocatalytic protocol, a wide range of similar enantioselective reactions were achieved with various radical sources, such as trifluoroborate,^148^ silanes^149^ and redox-active esters.^150^

### 4.3. C–O cleavage

C–O cleavage exemplified a typical reaction outcome of SET to generate R˙ from alcohol and its activated derivatives. Enlightened by the well-known Barton–McCombie deoxygenation with xanthate and its later generations,^151^ recent reports revealed various benign reaction conditions using photocatalysis.

**Scheme 35:**
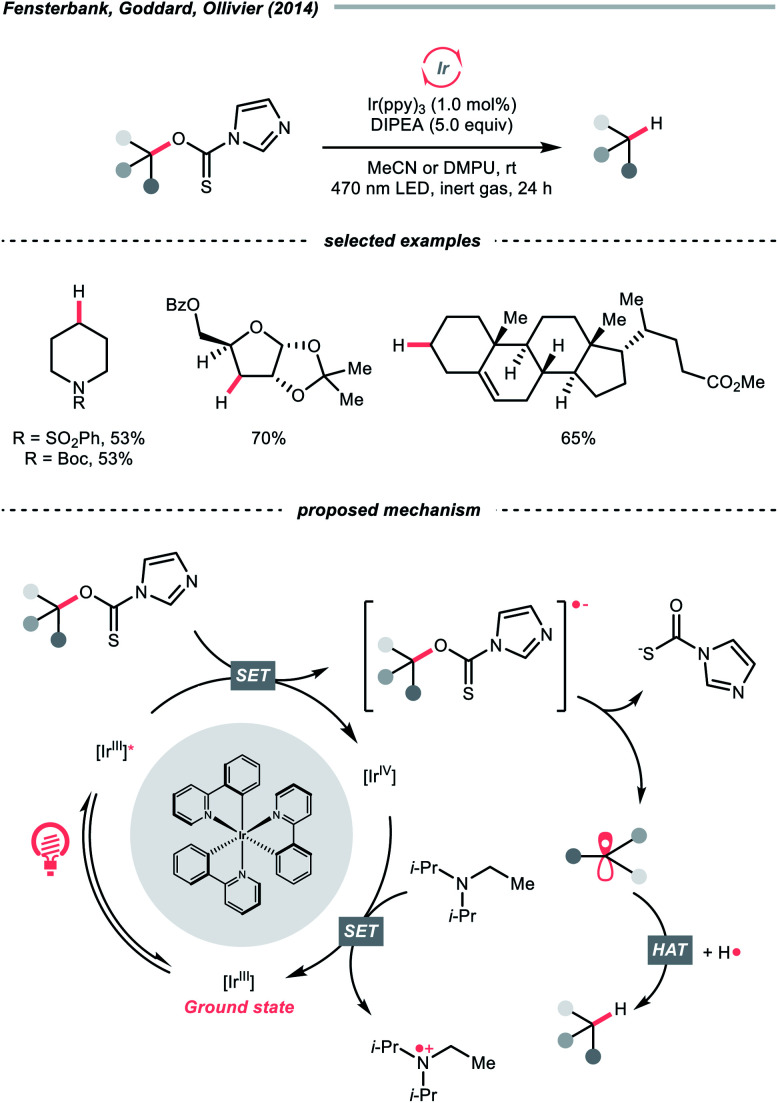
Photocatalytic deoxygenation of *O*-thiocarbamates.

In 2014, a photocatalysed tin-free Barton–McCombie-type deoxygenation reaction was demonstrated by the collaboration between Fensterbank's, Goddard's and Ollivier's group (Scheme 35).^152^ In the plausible mechanism, the *O*-thiocarbamate, (*E*^red^_1/2_ −1.56 to −1.73 V *vs.* SCE in MeCN) was reduced by the photoexcited Ir(ppy)_3_ (*E*^red^_1/2_ Ir(iv)/Ir*(iii) = −1.73 V *vs.* SCE in MeCN), followed by C–O cleavage to afford an R˙. The *N*,*N*-diisopropylethylamine (DIPEA) served not only as a reductant to reduce the Ir(iv) back to Ir(iii) but also as a hydrogen atom source to deliver the deoxygenated product.

**Scheme 36:**
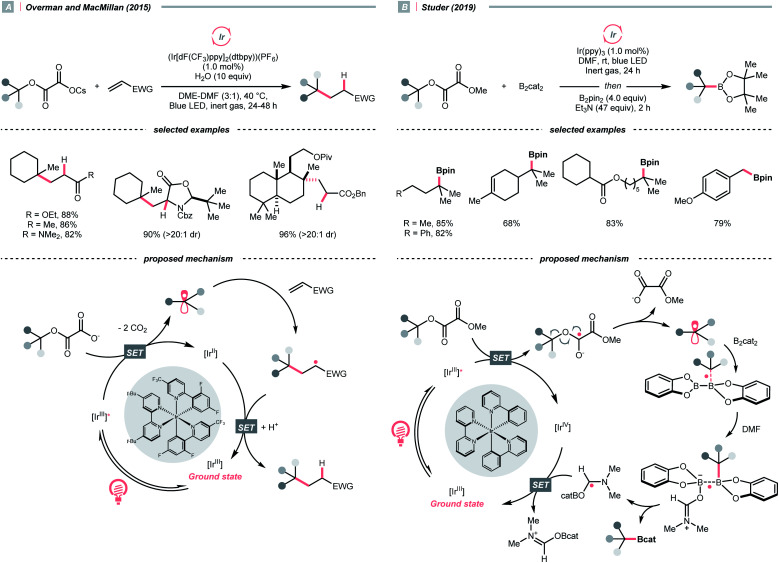
R˙ generation *via* C–O cleavage of oxalates.

Pertinent to the RAEs derived from carboxylic acids, Overman's group unveiled a similar *N*-phthalimidoyl oxalate as the activated alcohol and successfully applied it in the photocatalytic Giese-type approach to construct quaternary carbon centres.^153^ Due to the lability of such oxalate starting materials that led to purification issues, in 2015, the groups of Overman and MacMillan collaboratively advanced a novel protocol using alkali oxalate as the activating group, which possessed opposite redox property relative to the *N*-phthalimidoyl ones (Scheme 36A).^154^ In their new redox-neutral Giese reaction, reductive quenching of the Ir*(iii) (*E*^red^_1/2_ Ir*(iii)/Ir(ii) = +1.21 V *vs.* SCE in MeCN) by tertiary alcohol-derived cesium oxalate (for *tert*-BuOCOCO_2_Cs, *E*^red^_1/2_ = +1.28 V *vs.* SCE in MeCN) was conceived feasible due to the close reduction potential and the facile evolution of two CO_2_ molecules. The R˙ obtained from the C–O cleavage was added toward electron-deprived alkene, furnishing the alkylated product through electron and proton transfer with the rest of the photocatalytic cycle. To be noticed, the double decarboxylation of secondary alkali metal oxalates was less efficient, which slightly limited the scope of applicable alcohols. However, such an oxalate-based approach opened chemical space for various radical transformations soon after, as the same group published a metallaphotoredox cross-coupling reaction with the oxalate salts and aryl halides.^155^ Enlightened by these elegant examples, Wu's group disclosed a sodium xanthate-based photocatalysed Giese reaction, in which stoichiometric phosphine behaved as sacrificial reductant and sulfur transfer reagent.^156^

In contrast to the anionic oxalates, neutral dialkyl oxalate esters feature different redox properties, which could oxidatively quench the excited Ir(iii)-photocatalyst. In 2019, Studer and his co-workers successfully applied such a reaction paradigm in a radical borylation, wherein the tertiary alcohols were converted to methyl oxalates and subjected to single-electron reduction-initiated C–O cleavage to give the R˙ (Scheme 36B).^157^ In the same work, the substrate scope was extended to secondary alcohols *via* the xanthates and under thermal-induced radical conditions with tris(trimethylsilyl)silane (TTMSS) and 2,2′-azobis(2-methylpropionitrile) (AIBN).

**Scheme 37:**
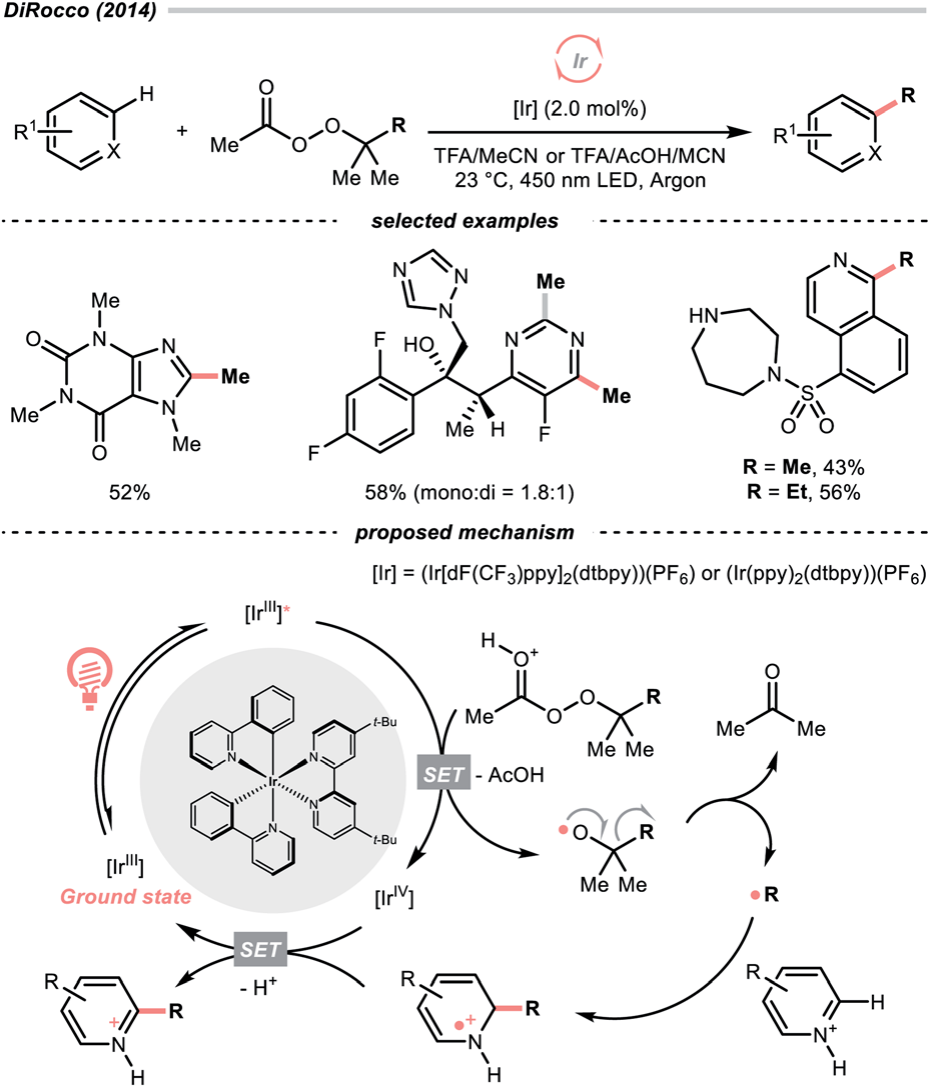
R˙ generation from peroxides.

While the abovementioned C–C cleavages with CO_2_ extrusion as the driving force, the group of DiRocco demonstrated a photocatalysed alkylation of bioactive heteroarenes by decomposing peroxides into R˙, with the formation of acetone as the driving force (Scheme 37).^158^ Using photoexcited Ir(iii) to reduce *tert*-butyl or *tert*-pentyl peracetate, *tert*-butoxy and *tert*-pentoxy radicals could be generated, respectively, which could undergo β-scission to deliver methyl and ethyl radicals for Minisci alkylations.

**Scheme 38:**
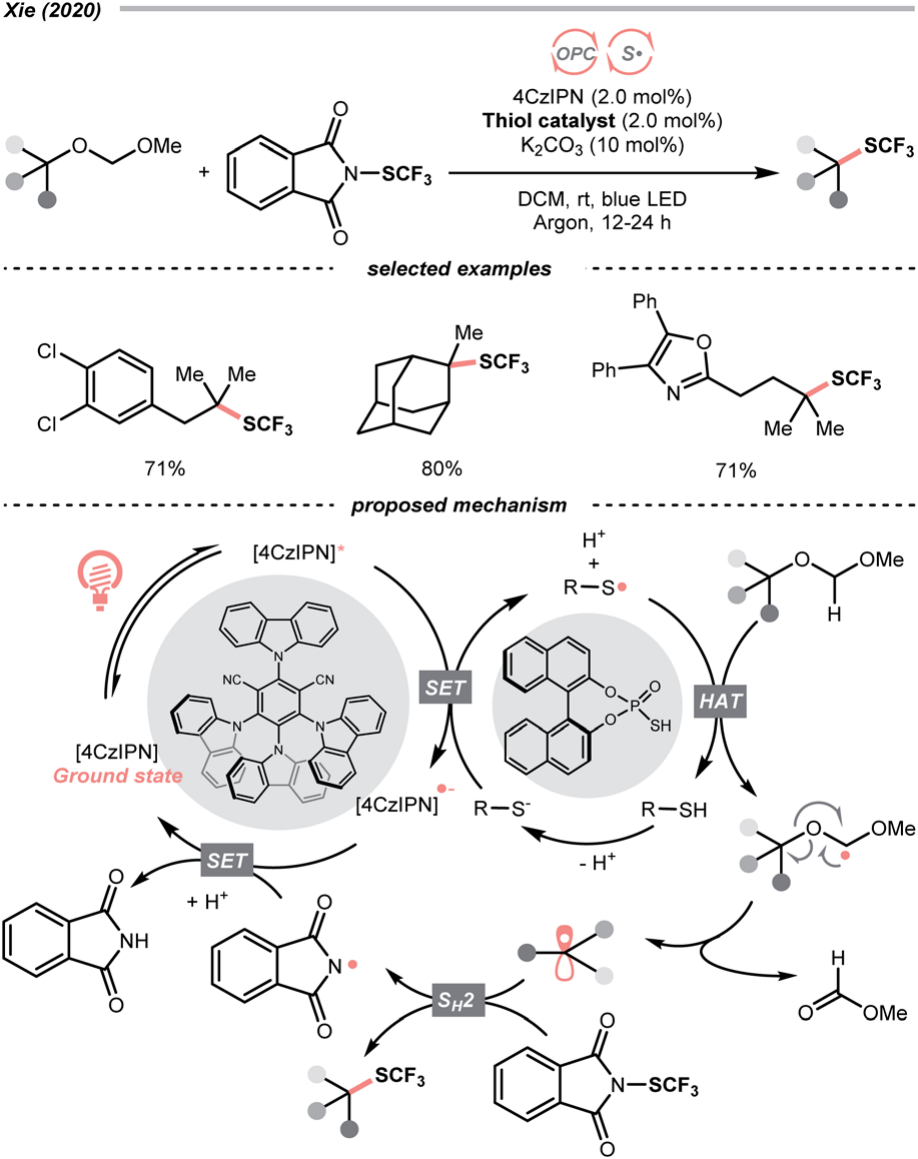
Using masked alcohol for R˙ generation.

Unlike the SET-based strategies used above, Xie *et al.* used dialkyl acetals as masked alcohols and conceived an HAT-enabled C–O trifluoromethylation reaction under a dual-photocatalytic manifold (Scheme 38).^159^ Mechanistically, the thiophosphate co-catalyst was firstly oxidised by the excited 4-CzIPN to form a thiyl radical, which performed a highly regioselective HAT at the acetal α-C–H position. The ensuing dialkoxy radical underwent β-C–O cleavage and turned into an R˙. The R˙ was then intercepted by the *N*-(trifluoromethylthio)phthalimide to deliver the aliphatic trifluoromethylthioether product. Although only tertiary alcohols were demonstrated in the scope, such an R˙-generation protocol were shown applicable in C–O fluorination in their later publication.^160^

**Scheme 39:**
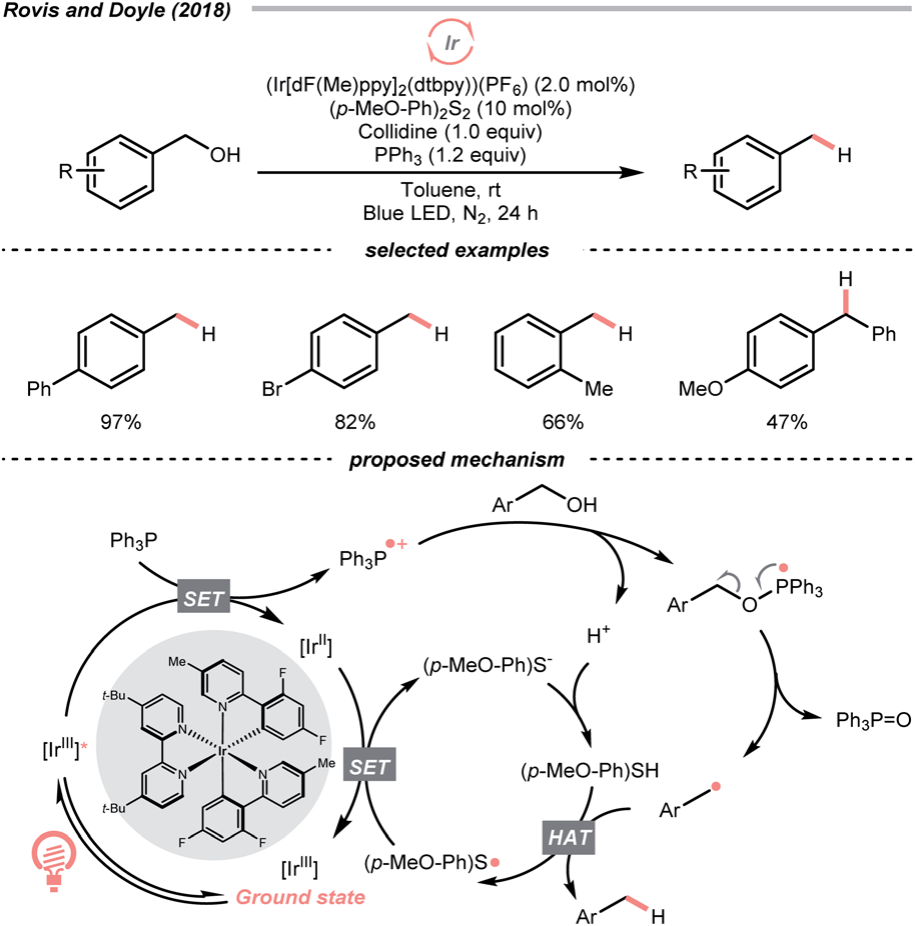
Direct hydrodeoxygenation of alcohols *via* R˙.

Interestingly, free alcohols could be directly utilised as alkylating reagents *via* deoxygenation. As shown by Doyle and Rovis *et al.*, under the iridium photocatalysis, phosphoranyl radical could be generated catalytically from the corresponding phosphine, which deoxygenated the free alcohol and released the R˙ (Scheme 39).^161^ Although this hydrodeoxygenative condition could only accommodate benzyl alcohols, it opened up new chemical space to efficiently extract alkyl radicals from alcohols.

### 4.4. C=O cleavage

**Scheme 40:**
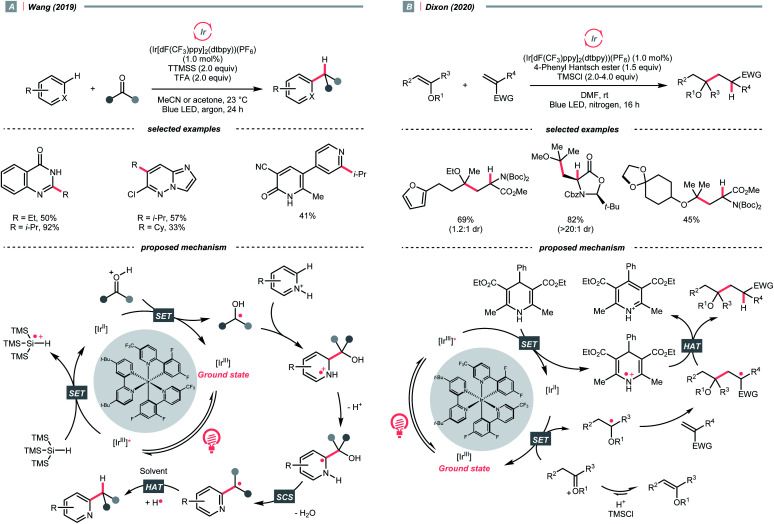
R˙ generation *via* carbonyl group reductions.

With photoredox catalysis, C=O could also act as an R˙ precursor *via* SET-enabled C=O cleavage. Combining [Ir(ppy)_2_(dtbbpy)]PF_6,_ TTMSS, and trifluoroacetic acid (TFA), Wang's group realised a photocatalysed deoxygenative hydroheteroarylation of ketone (Scheme 40A).^162^ A stepwise C–O cleavage mechanism involving ketyl radical (R˙) was proposed. Hypothetically, the protonated ketone obtained an electron *via* a PCET mechanism, which derived from the photoreduction of [Ir(iii)]* (*E*^red^_1/2_ Ir*(iii)/Ir(ii) = +1.21 V *vs.* SCE in MeCN) by TTMSS (*E*^red^_1/2_ = +0.73 V *vs.* SCE in MeCN). The nucleophilic ketyl radical was added to the protonated heteroarene, which gave an α-aminoradical after proton transfer. Driven by the rearomatisation, an SCS process proceeded with the removal of H_2_O, providing the alkylated heteroarene *via* the HAT with solvent. Concurrently, Huang's group reported a similar deoxygenative Minisci alkylation with aldehydes, which was proposed as a photocatalytic Br˙-mediated process.^163^

Carbonyl derivative or *in situ* generated carbonyl analogue are also viable precursors of R˙, while preserving the C–O bonds in products were typical reaction outcomes. In 2020, Dixon *et al.* utilised alkyl enol ethers under acidic conditions and generated oxonium tautomers *in situ* as activated carbonyl and R˙ precursor (Scheme 40B).^142^ In this work, the oxonium ion (calculated *E*^red^_1/2_ = −1.12 V *vs.* SCE in MeCN) was reduced by the Ir(ii) (*E*^red^_1/2_ Ir(iii)/Ir(ii) = −1.37 V *vs.* SCE in MeCN) derived from the SET between excited Ir(iii)-photocatalyst (*E*^red^_1/2_ Ir*(iii)/Ir(ii) = +1.21 V *vs.* SCE in MeCN) and 4-phenyl Hantzsch ester (calculated *E*^red^_1/2_ = +0.75 V *vs.* SCE in MeCN), forming an α-ethereal radical (R˙). Adding such an R˙ to the conjugated alkenes followed by HAT with the Hantzsch ester brought the α-tertiary dialkyl ethers as desired products.

### 4.5. C–S cleavage

Due to the flexible oxidation state of sulfur, various *S*-based radical alkylating reagents were developed, accompanied by their photocatalytic systems.

**Scheme 41:**
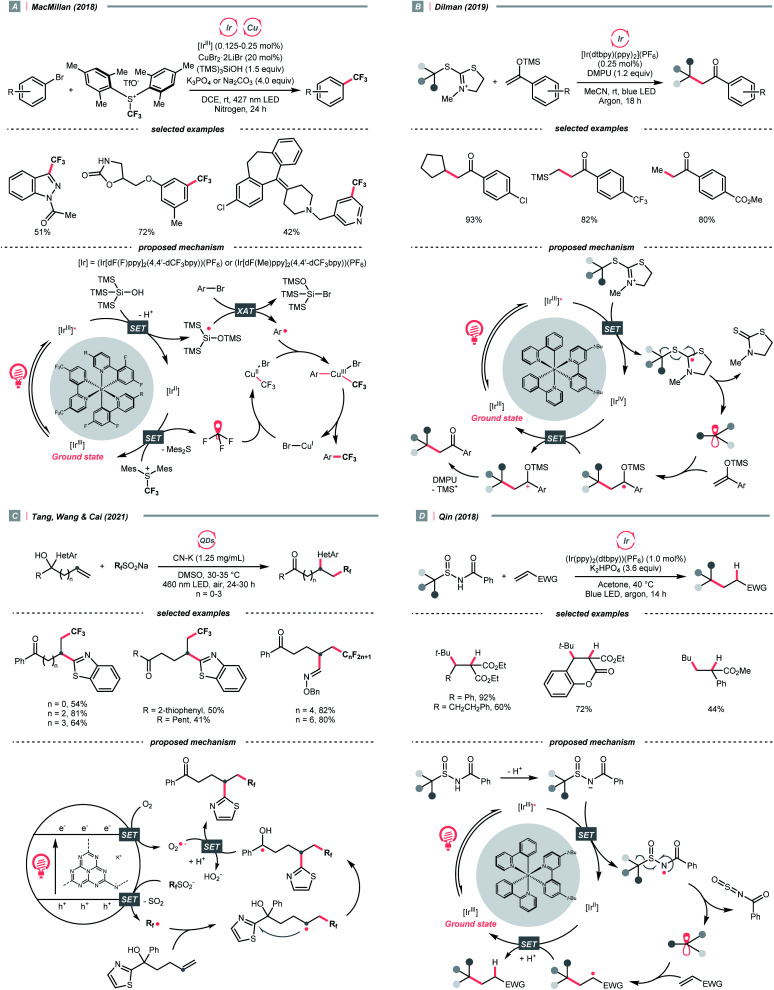
R˙ from sulfoniums, thiazoliniums, sulfinates and sulfinamides.

Alkyl sulfonium salt can be easily synthesised from alkyl halides, thiols and alcohols.^164–168^ Upon receiving an electron, such a trivalent species undergoes R–S cleavage to release an R˙ and a thioether. In 2018, MacMillan *et al.* disclosed a Cu(ii)/Ir(iii) dual photocatalytic system to couple aryl bromides and trifluoromethylsulfonium salt in the presence of a supersilanol (Scheme 41A).^169^ The photoredox cycle began with the generation of Si˙ *via* SET (*E*^red^_1/2_ Ir*(iii)/Ir(ii) = +1.55 V *vs.* SCE in MeCN; for (TMS)_3_SiOH, *E*^red^_1/2_ = +1.54 V *vs.* SCE in MeCN). The aryl bromide was subjected to Si˙-mediated halogen atom transfer (XAT, see Section 4.6), giving the aryl radical (Ar˙). On the other hand, interaction between modified Umemoto's reagent, dimesityl(trifluoromethyl)sulfonium (dMesSCF_3_ or MacMillan's trifluoromethylation reagent, *E*^red^_1/2_ = −0.52 V *vs.* SCE in MeCN) and Ir(ii) (*E*^red^_1/2_ Ir(iii)/Ir(ii) = −0.83 V *vs.* SCE in MeCN) gave 
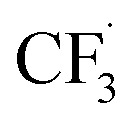
 through reductive C–S cleavage. On the copper side, the interception of 
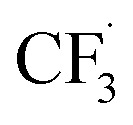
 by Cu(i) preceded the Ar˙ trapping, and the facile Cu(iii) reductive elimination rendered the trifluorotoluenes as desired products. The same tactic could apply to alkyl bromides, as shown in their later report.^170^

With the same Ir-photocatalyst for SET reduction, Dilman *et al.* exploited the ketone synthesis using dihydrothiazolinium salts as R˙ precursors, which could be simply prepared from alkyl bromides (Scheme 41B).^171^ Upon being reduced by the photoexcited Ir(iii), dihydrothiazoline radical fragmented into an R˙ along with a thione byproduct. Silyl enol ether trapped the R˙, followed by oxidation and silyl group removal to give the ketone product.

Instead of SET reduction, sulfinate and sulfinamide are other versatile S-containing radical alkylating reagents that give R˙ upon oxidative decomposition. In 2021, an application of perfluoroalkyl sulfinates (NaSO_2_R_f_) in migratory alkene difunctionalisations with intramolecular heteroaryl groups was presented by the research team of Tang, Wang, and Cai (Scheme 41C).^172^ Using potassium-modified carbon nitride (CN–K) as a recyclable photosensitive material, the electron–hole pair generated under light irradiation and ambient atmosphere could oxidise NaSO_2_R_f_ into a perfluoroalkyl radical 
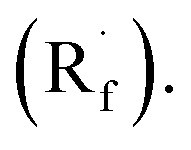
 The 
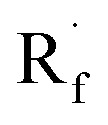
 addition to the olefin tail triggered the heteroaryl group migration, which furnished the terminally perfluoroalkylated ketone as the expected product after losing a proton and electron. Interestingly, formyl and benzyl oxime ether groups can undertake the same type of functional group translocation.

In 2018, Qin *et al.* documented a desulfurative R˙ generation strategy with *N*-benzoyl alkylsulfinamide using Ir-photoredox catalysis (Scheme 41D).^173^ As in typical oxidation-initiated Giese reaction, the R˙ was produced by the SET between Ir*(iii) (*E*^red^_1/2_ Ir*(iii)/Ir(ii) = +1.21 V *vs.* SCE in MeCN) and deprotonated sulfinamide (for neutral *tert*-butyl sulfinamide, *E*^red^_1/2_ = +0.66 V *vs.* SCE in MeCN), which was accompanied by a C–S bond cleavage to give the R˙ and *N*-sulfinylbenzamide byproduct. By performing the R˙ conjugate addition to electron-deficient alkenes, reactions with secondary and tertiary alkylsulfinamides provided alkylated products with generally good to excellent yields, while primary ones were less efficient.

Beyond the SET territory, homolytic cleavage of C–S bonds to give R˙ could be realised under visible light irradiation. However, this kind of bond-breaking pattern remained rare in practical synthesis, especially for intermolecular transformations, since controlling the reaction outcome with a radical pair could be challenging. Also, these twin radicals have more tendency to recombine or undergo side reactions, which could undermine the effective concentration of desired R˙. In light of these difficulties, the strategic introduction of a photocatalyst that could mediate the reactivities of both radicals was crucial to the success of two-component or even multi-component radical reactions.

**Scheme 42:**
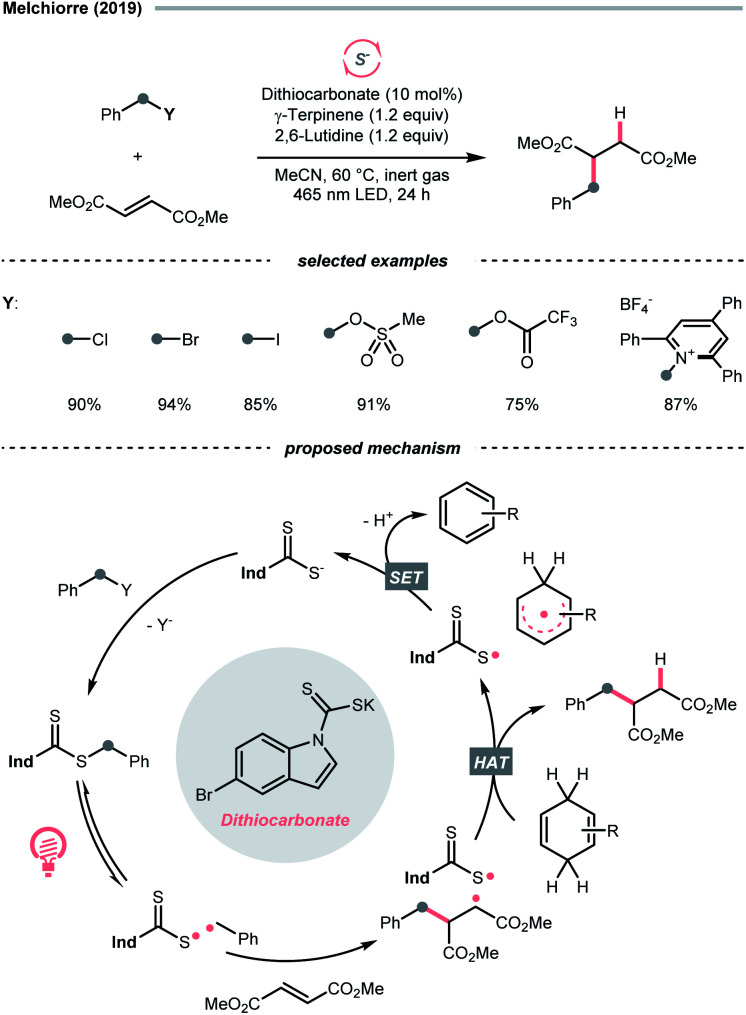
Photocatalytic R˙ generation *via* R–S homolysis.

In 2019, the group of Melchiorre designed an elegant indole-based dithiocarbamate organocatalyst to tackle this challenge (Scheme 42).^174^ In their proposed mechanism, the nucleophilic attack of the dithiocarbamate to alkyl halides/pseudohalides could form a visible light-absorbing species, which could undergo R–S bond homolysis to afford a thiyl radical and the desired R˙. With a substoichiometric quantity of dithiocarbamate as a photochemical trigger, the σ-bond homolysis to give R˙ becomes much more efficient and controllable. To this end, R˙ could be engaged in the conjugate addition with electron-deficient alkenes smoothly (Giese addition), wherein the γ-terpinene was added as the terminal reductant, serving as the product hydrogen atom source and for catalyst turnover. It was noteworthy that the side reactions with thiyl radical were inconsequential since the so-formed adduct could be resubjected to the light-enabled homolysis and liberate the thiyl radical for its catalytic cycle. Soon after, this organocatalytic photochemical R˙ generation method was embedded in many other alkylative transformations, including alkene difunctionalisations, two- and three-component aromatic alkylation,^175^ and borylation.^176^

### 4.6. C–Br and C–I cleavages

Alkyl halides are among the earliest employed R˙ precursors and still widely used in modern synthesis because of their structural diversity and commercial availability. Among them, iodides and bromides, which possess relatively weak C–Br and C–I bonds, are more popular choices than the corresponding chlorides and fluorides.^177–181^ Although many photochemical alkylation reactions *via* homolytic cleavage of weak C–I bond were precedented, they often involve direct irradiation with UV light, leading to narrow substrate scope and less controllable radical process. Gratifyingly, recent advancements have demonstrated that this type of R˙ generation conditions could be significantly improved under visible light irradiation with the aid of photocatalysts.^182^

**Scheme 43:**
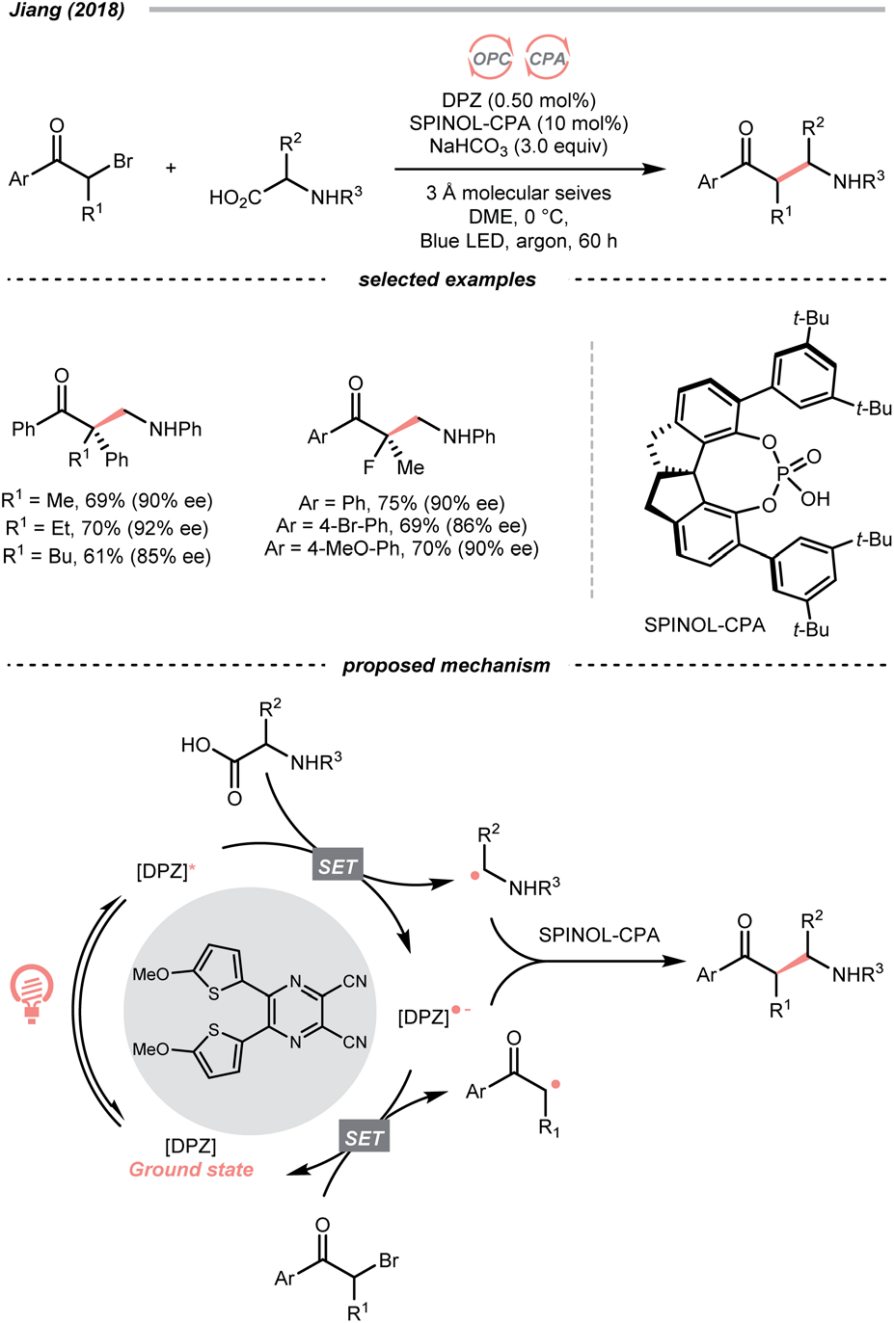
Employing alkyl bromides as R˙ precursors.

The rapid progress of photoredox chemistry endows alkyl halides with new activation modes, among which SET represents the mostly seen ways to streamline the generation of R˙.^183^ In 2018, Jiang *et al.* disclosed an elegant enantioselective cross-coupling between α-bromoketones and α-amino acids with the aid of chiral phosphoric acid (SPINOL-CPA) and dicyanopyrazine-derived chromophore (DPZ) under visible light irradiation (Scheme 43).^184^ The photoexcited DPZ functioned as an electron transfer catalyst, which mediated the generation of two electronically distinct R˙ from the alkyl bromide and carboxylic acid *via* reductive and oxidative SET, respectively. The CPA then managed the radical–radical cross-coupling with these two R˙ in an enantioselective fashion, synthesizing numerous β-amino ketones in good to excellent enantioselectivities.

**Scheme 44:**
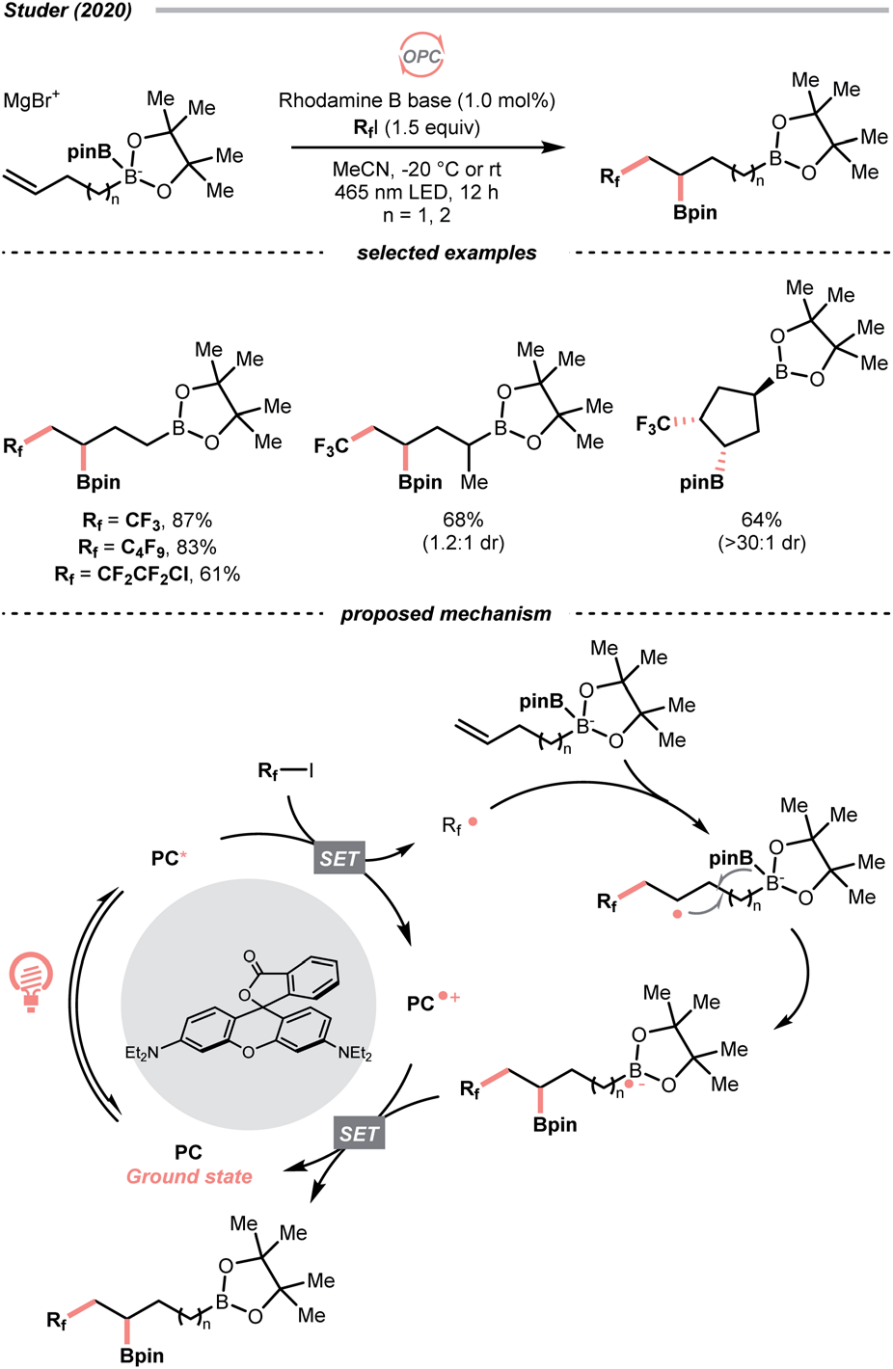
R˙ formation from alkyl iodide.

For iodides, in 2020, Studer's laboratory showcased a synthetic approach toward perfluoroalkylated diborylalkanes using perfluoroalkyl iodides and anionic diboron complexes, which were formed by *in situ* mixing Grignard reagent and bis(pinacolato)diboron (B_2_pin_2_) (Scheme 44).^185^ Similar to the alkyl bromides above, the perfluoroalkyl iodide (*E*^red^_1/2_ = −1.52 V *vs.* SCE in DMF for CF_3_I) received an electron from the excited Rhodamine B, forming an 
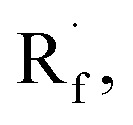
 which combined with an alkene to give a new R′˙. Distal boron migration with the intramolecular Bpin group following single-electron oxidation delivered the desired 1,n-bisborylalkane products (n = 3 and 4).

Besides the SET pathway to form R˙ from alkyl halides, halogen atom transfer (XAT) has demonstrated its synthetic utility for decades. Historically, XAT was enabled by some metallic radicals (*e.g.*, tin, chromium, cerium, gold) that can abstract halogen atoms from alkyl halides and give R˙.^186–190^ With the surging development of photocatalysis, various new XAT agents could be accessed efficiently under mild photocatalytic conditions, especially with those non-metallic ones such as silyl and C-centred radicals, which significantly broaden the application of such an R˙-generating strategy.^190^

**Scheme 45:**
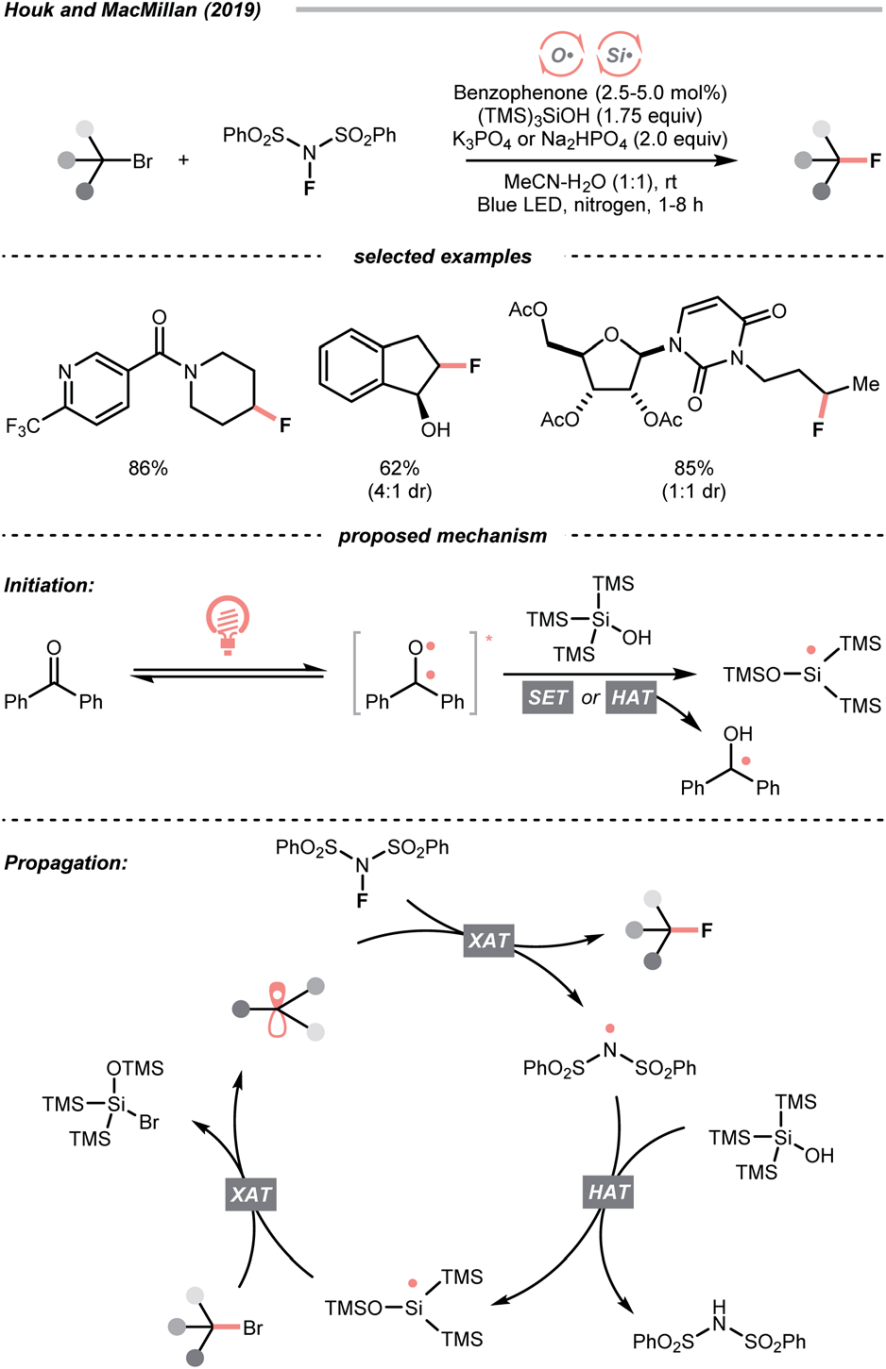
Silyl radical-mediated XAT for R˙ generation.

In this context, MacMillan and Houk's team developed a photoinduced reductive fluorination reaction of alkyl bromide *via* the XAT process (Scheme 45).^191^ Based on the supersilanol-mediated bromine atom transfer strategy developed in MacMillan's laboratory,^192–194^ this reaction was optimised with catalytic benzophenone and stoichiometric quantity of silyl radical source, (tris(trimethylsilyl)silanol (TMS)_3_SiOH) and electrophilic fluorinating reagent, *N*-fluorobenzenesulfonimide (NFSI). Initially, under blue LED irradiation, the excited benzophenone catalysed the generation of silyl radical (Si˙), in which the supersilanol might experience an HAT or SET process followed by the Brook rearrangement. Then, the Si˙ was subjected to the XAT with the alkyl bromide to give R˙. The R˙ was fluorinated by NFSI to produce an alkyl fluoride and an NCR (sulfonamido radical), which could regenerate the Si˙ and propagate the radical chain process.

**Scheme 46:**
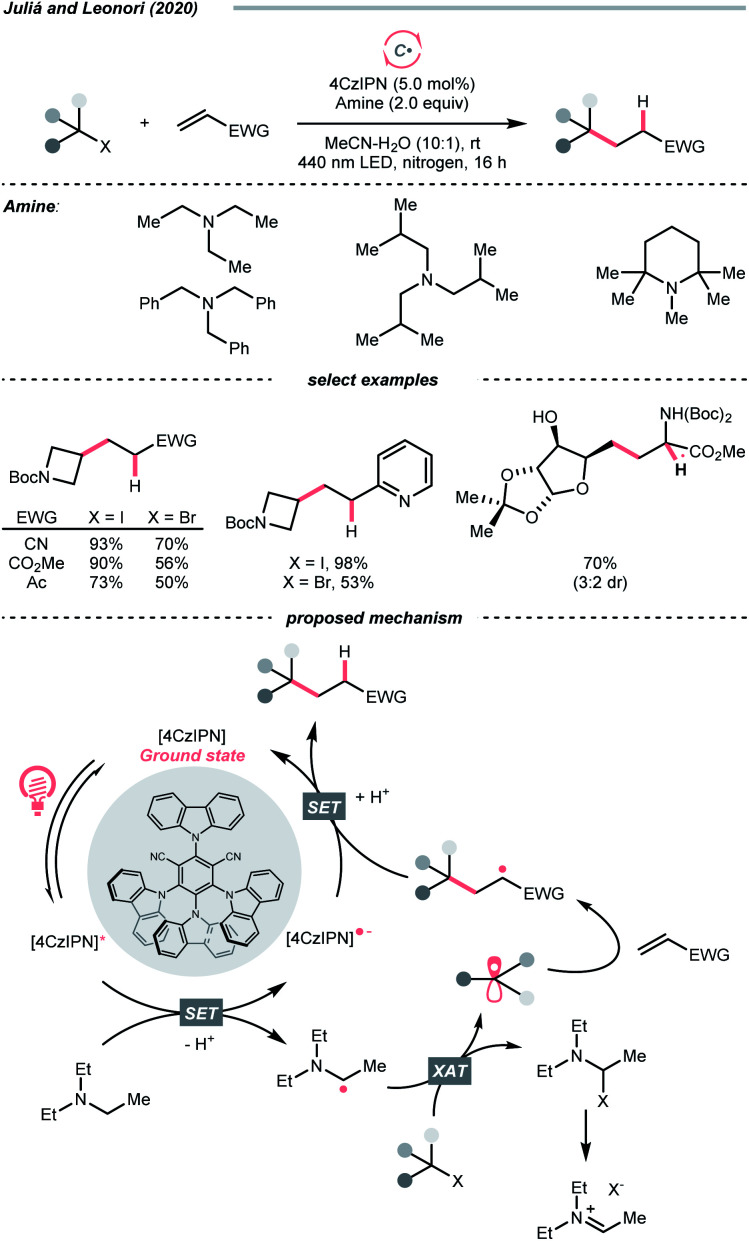
Tertiary amine-mediated XAT for R˙ formation.

In addition to Si˙, using C-centred radicals for XAT with alkyl halides was recently demonstrated by Juliá, Leonori and their co-workers. In this contribution, a C(sp^3^)–C(sp^3^) bond formation reaction between alkyl halides and alkenes was disclosed, which was mediated by organophotoredox catalyst 4CzIPN under visible light irradiation (Scheme 46).^195^ Initially, an α-amino radical resulted from the single-electron oxidation of tertiary amine by excited 4CzIPN. The key XAT benefited from the strong nucleophilicity of α-aminoalkyl radicals, which stabilised the polar XAT transition state and facilitated the R˙ generation. Then, the R˙ followed the typical reaction pathway of Giese-type radical addition to alkenes. A judicious choice of XAT reagent is essential in this design since the XAT byproduct, α-iodoamine, could degrade into an iminium iodide, therefore, combating the back halogen atom transfer.

### 4.7. C–B cleavage

**Scheme 47:**
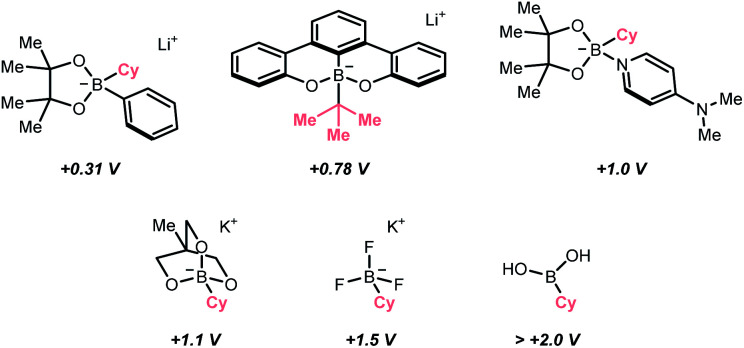
Common boron-based R˙ precursors.

Like the alkyl halides, boronic acid and its derivatives are highly enabling R˙ precursors, however, featuring opposite electronic demand during the SET with photoredox catalysts (Scheme 47). Among them, potassium trifluoroborate, which was intensively studied by Molander's and other groups, is a common option due to its high shelf stability and easily accessible redox potential.^196–206^

**Scheme 48:**
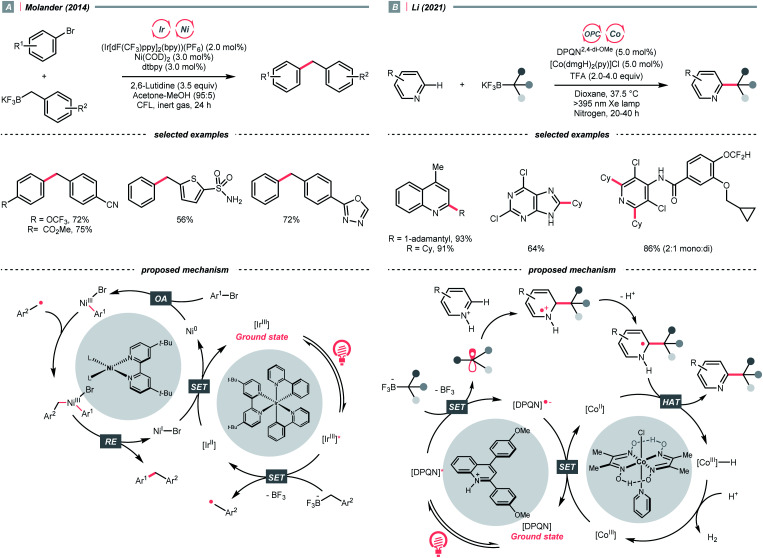
Alkyl trifluoroborate as R˙ source.

By merging photocatalysis and nickel catalysis, the challenging C(sp^2^)–C(sp^3^) Suzuki–Miyaura coupling with aryl bromides and benzyltrifluoroborates was realised by Molander's group in 2014 (Scheme 48A).^207^ Soon after, secondary,^208^ tertiary^209^ trifluoroborates and other variants are shown as effective in their later publications. The SET between Ir*(iii) (*E*^red^_1/2_ Ir*(iii)/Ir(ii) = +1.32 V *vs.* SCE in MeCN) and trifluoroborate (for the potentials of R–BF_3_K *vs.* SCE in MeCN, R = Bn, *E*^red^_1/2_ = +1.10 V; R = Cy, *E*^red^_1/2_ = +1.50 V; R = *tert*-Bu, *E*^red^_1/2_ = +1.26 V) and the trapping of corresponding R˙ by Ar–Ni(ii) to promote the product-forming reductive elimination were two common mechanistic traits for all these dual catalysed cross-couplings. Unlike the conventional polar transmetalation pathways in which alkyl nucleophiles were often problematic organometallic partners, such a single-electron scenario with alkyl trifluoroborates grants unique and complementary reactivities under the radical mechanism.

Instead of metallaphotocatalysis, organophotoredox catalysts that were highly oxidising at the excited state could also pair with the alkyl trifluoroborate to effect R˙ generation. In 2021, Li's group reported a quinolinium/cobaloxime co-catalysed Minisci alkylation of alkyl trifluoroborates without external chemical oxidant (Scheme 48B).^210^ With detailed mechanistic studies, they conceptualised a proton-activation mode of *N*-heteroaromatics and developed a novel 2,4-bis(4-methoxyphenyl)quinoline organophotocatalyst (DPQN^2,4-di-OMe^) with an extensive oxidation window (
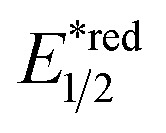
 = +1.96 V *vs.* SCE in MeCN).^62^ SET with different alkyl trifluoroborates broke the C–B bonds and turned them into R˙, which was enrolled in the typical Minisci alkylation mechanism. Strategically, a cobalt(iii) catalyst was introduced to drive this transformation and balance its redox equation *via* H_2_ evolution. This novel organophotocatalyst could work with many other R˙ precursors, *e.g.*, Hantzsch ester, sulfinate, hydrazide, dihydrobenzothiazoles, bicyclo[2.2.0]hexene as well as oxalic acid. The same group also showed that other types of oxidative alkylations with alkenes and alkyne could be achieved, and the immobilised quinolinium photocatalyst on polymeric support could be recycled multiple times.

Interestingly, capitalizing on the unique solvent effect of *N*,*N*-dimethylacetamide (DMA), Sharma's group tackled the intractable one-electron activation of free boronic acids, realizing various R˙-involving processes *via* C–B bond cleavage.^211^

**Scheme 49:**
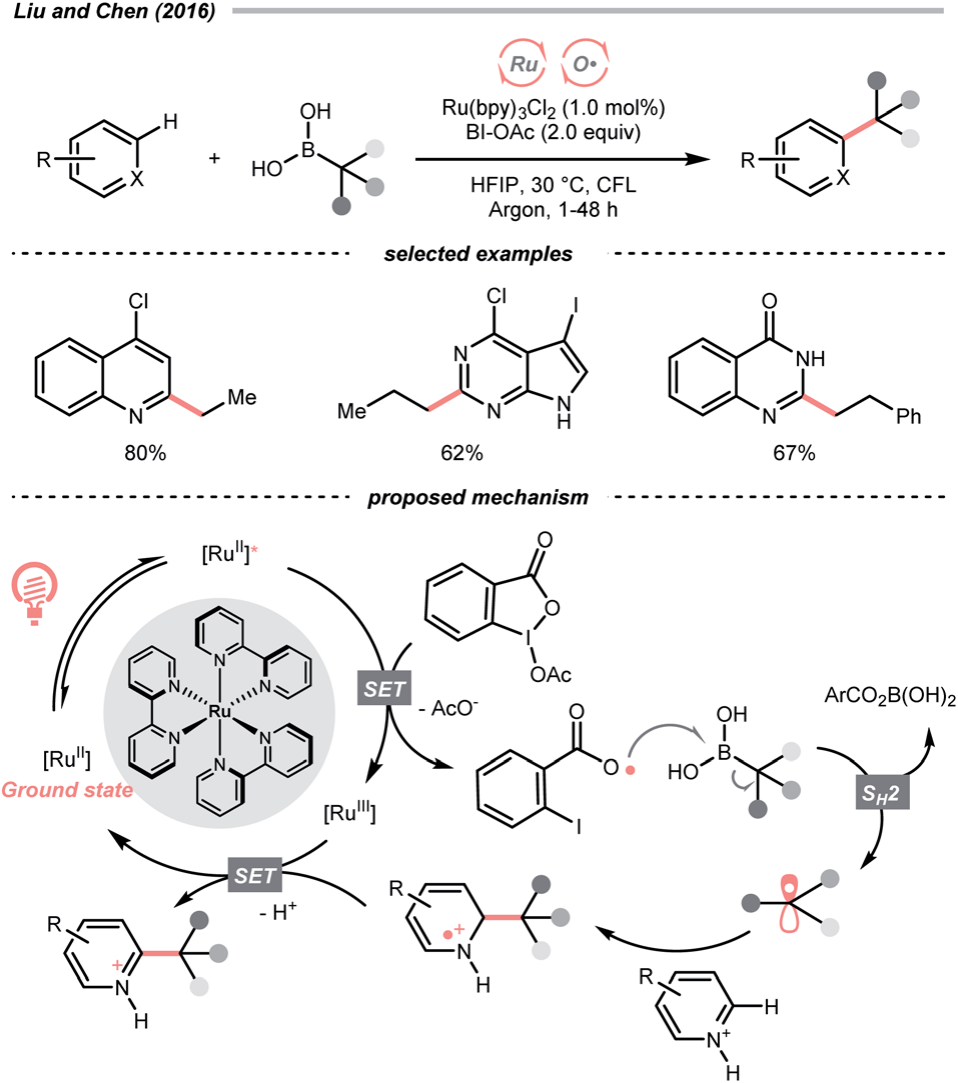
R˙ formation *via* S_H_2 of alkyl boronic acid.

In addition to the single-electron oxidation of boronic acids or boronates, S_H_2 of aliphatic boronic acids to extrude R˙ was also operative. In 2016, Liu and Chen's team reported a photocatalytic Minisci alkylation with alkyl boronic acids in the presence of BI-OAc oxidant and ruthenium(ii) photocatalyst (Scheme 49).^212^ The photoexcited Ru(ii) firstly reduced BI-OAc to 2-iodobenzoyloxy radical. Counting on the B–O affinity, the oxy radical addition to boronic acid would induce the C–B cleavage and release an R˙, which was supported by the DFT calculations. The R˙ was then subjected to the heteroarene alkylation.

### 4.8. C–Si cleavage

**Scheme 50:**
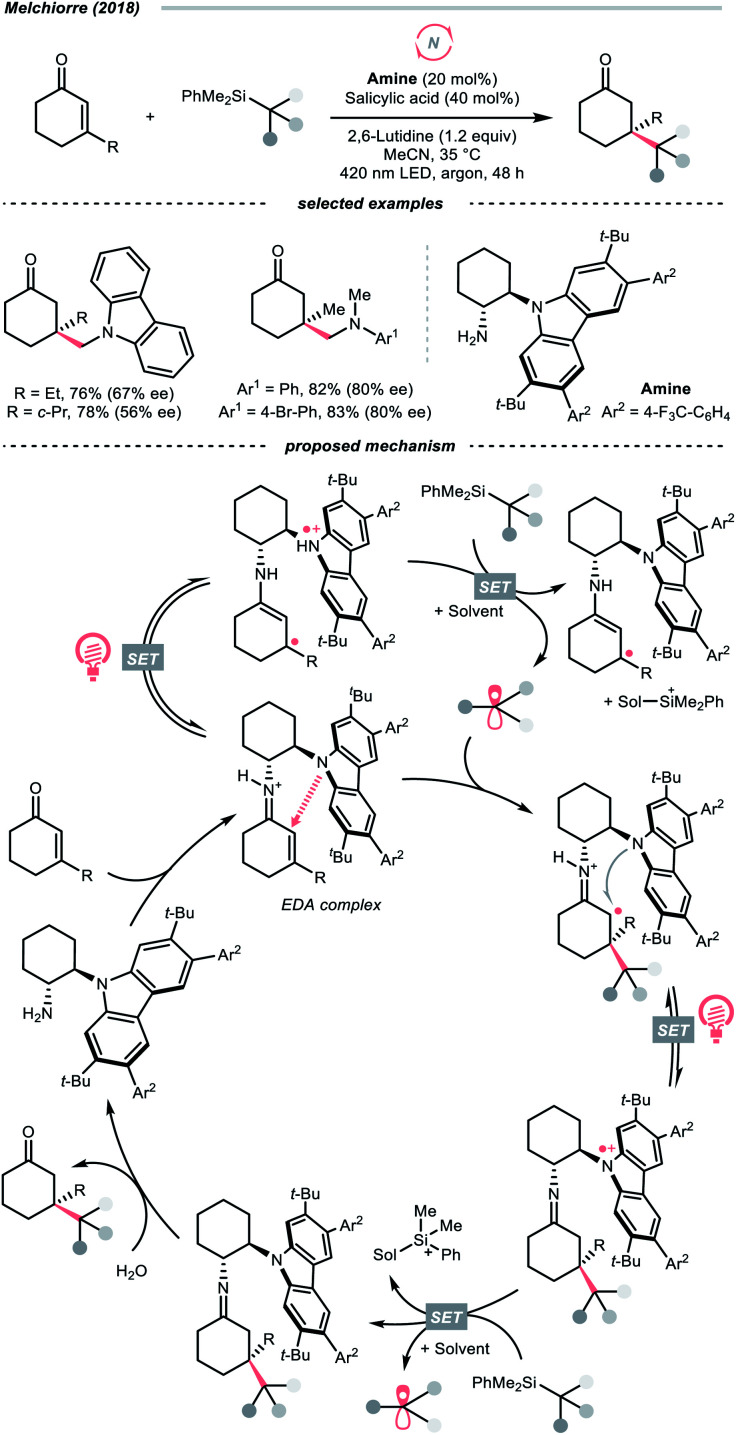
Oxidation of alkyl silane for R˙ formation.

Analogous to boronic acids, silane and other silica-based compounds are organometallic reagents that effect the R˙ generation by C–Si bond scission under mildly photoredox conditions. One application of alkylsilane in asymmetric addition to α,β-unsaturated ketones was demonstrated by Melchiorre's laboratory in 2018 (Scheme 50).^213^ Combining the elegant iminium-catalysed photooxidation^214^ and electron-donor acceptor (EDA) chemistry established in his group,^215^ a chiral carbazole-tethered amine organocatalyst was developed. The iminium EDA intermediate formed by condensing the amine catalyst and conjugated ketone absorbed visible light and enabled an intramolecular electron transfer to create a long-lived carbazole radical cation (*E*^red^_1/2_ = +1.11 V *vs.* SCE in MeCN). Silanes within this redox window, typically those α-nitrogenated, were applicable R˙ precursors, which would undergo an oxidative fragmentation to break the C–Si bonds. Importantly, the MeCN coordination could facilitate the desilylation by forming a [MeCN-SiR_3_]^+^ complex and inhibiting back-electron transfer.

The carbazole handle was bifunctional. On the one hand, guided by such a bulky shield, R˙ will attack the conjugated iminium preferentially at the less hindered face, giving an α-iminyl radical. On the other hand, the electron-rich carbazole could be an electron sink; therefore, another intramolecular SET will give a new carbazole radical cation, which substantiated a radical chain mechanism. Subsequent hydrolysis of the β-functionalised imine led to product formation and catalyst regeneration. It is notable that recently, the same group reported an asymmetric C–C cross-coupling between aliphatic silanes and allylic alcohols, in which the photoexcitable allylic Ir(iii) complex mediated the R˙ formation and the chiral induction for the C–C bond-forming step.^216^

As explored in Fensterbank's and Kano's studies and others, anionic hypervalent silicate could generate the R˙ under comparably milder conditions due to its anionic character. On this basis, two different types of silicates were applied in photoredox chemistry.

**Scheme 51:**
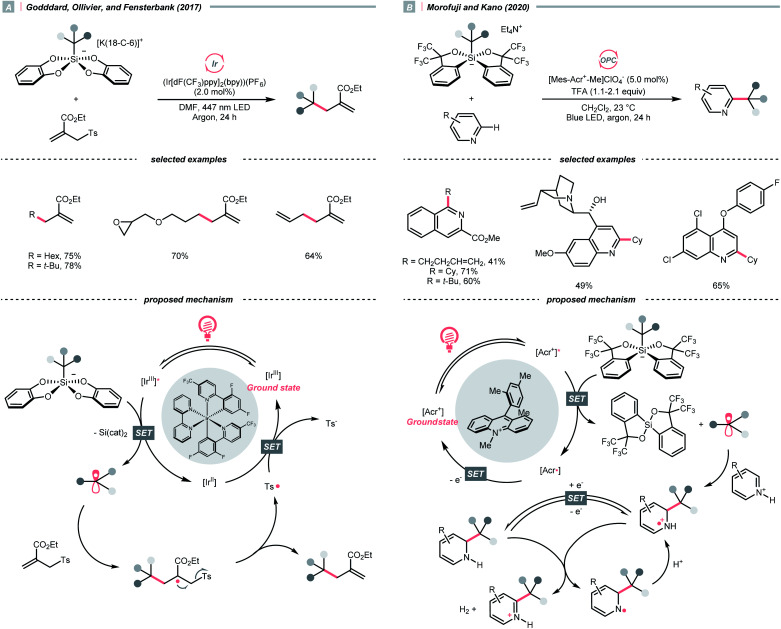
Silicate-based reagents as R˙ precursors.

In 2017, Fensterbank, Ollivier, Goddard and their co-workers collaboratively disclosed a photocatalysed radical alkylation reaction *via* oxidation of biscatecholato silicates (Scheme 51A).^217^ A series of silicates were prepared *via* modular synthesis, which showed accessible reduction potentials (for [CySi^−^(cat)_2_][K^+^(18-C-6)], *E*^red^_1/2_ = +0.69 V *vs.* SCE in DMF) by the commercially available Ir(iii)-photocatalyst (*E*^red^_1/2_ Ir*(iii)/Ir(ii) = +1.32 V *vs.* SCE in MeCN). The R˙ resulting from the C–Si cleavage could be employed in Giese-type addition or allylation reaction *via* the S_H_2 mechanism. Additionally, the versatility of this type of silicate reagent could be extended to cross-couplings with nickel catalysis.^218,219^

However, when biscatecholato silicates were applied to Minisci-type alkylation, the reaction efficiency was largely compromised by their decomposition under acidic conditions. Stepping forward, Kano *et al.* devised a new acid-stable pentavalent silicate reagent (for cyclohexyl one, *E*^red^_1/2_ = +1.47 V *vs.* SCE in MeCN), which could generate the desired R˙ for heteroarene alkylation after being oxidised by [Mes-Acr-Me]^+^ClO_4_^−^ (
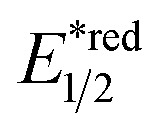
 = +2.06 V *vs.* SCE in MeCN, Scheme 51B).^220^ Interestingly, with the [Mes-Acr^+^-Ph]BF_4_^−^ photocatalyst (
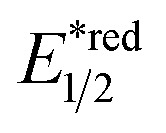
 = +2.08 V *vs.* SCE in MeCN), radical addition to electron-deficient alkenes was also achieved by the same group, including methylation.^221^

## 5. Conclusions

C(sp^3^) radicals have been increasingly serving as aliphatic functionalising species in organic synthesis, and photocatalysis represents one of the most advanced approaches to enable the C(sp^3^) radical generation and the subsequent radical functionalisations in recent years.

This review listed some typical contributions in this field and classified them based on types of bond cleavage and the corresponding R˙ generation strategies. Along this line, distinguished mechanistic traits of these radical C–H, C–C and C–X functionalisation examples were analysed, discussed and compared in detail. By summarising these works, we wish to offer the readers a systematic overview of this exciting area and, more importantly, inspire future research endeavours.

We are optimistic that photocatalytic examples that provide more and more bond-breaking and -forming opportunities will appear in the literature. Especially, those incorporating some interdisciplinary techniques, *e.g.*, photoelectrochemistry and biocatalysis, would pronouncedly enrich the chemist's toolkit for C(sp^3^)-based skeleton synthesis. Moreover, we believe that these established C(sp^3^) radical logic could be extended, effecting the generation of other C-centred radicals such as aryl, vinyl and alkynyl radicals.

## Author contributions

The manuscript was written through the contributions of all authors. All authors have given approval to the final version of the manuscript.

## Conflicts of interest

There are no conflicts to declare.

## Supplementary Material
